# Transcription organizes euchromatin via microphase separation

**DOI:** 10.1038/s41467-021-21589-3

**Published:** 2021-03-01

**Authors:** Lennart Hilbert, Yuko Sato, Ksenia Kuznetsova, Tommaso Bianucci, Hiroshi Kimura, Frank Jülicher, Alf Honigmann, Vasily Zaburdaev, Nadine L. Vastenhouw

**Affiliations:** 1 Center for Systems Biology Dresden, Dresden, Germany; 2 Max Planck Institute of Molecular Cell Biology and Genetics, Dresden, Germany; 3 Max Planck Institute for the Physics of Complex Systems, Dresden, Germany; 4 Tokyo Institute of Technology, Yokohama, Kanagawa Japan; 5 Center for Advancing Electronics Dresden, Technical University Dresden, Dresden, Germany; 6 Cluster of Excellence Physics of Life, Technical University Dresden, Dresden, Germany; 7 Present Address: Institute of Biological and Chemical Systems, Karlsruhe Institute of Technology, Eggenstein-Leopoldshafen, Germany; 8 Present Address: Zoological Institute, Karlsruhe Institute of Technology, Karlsruhe, Germany; 9 Present Address: Friedrich-Alexander Universität Erlangen-Nuremberg, Max Planck Zentrum für Physik und Medizin, Erlangen, Germany; 10 Present Address: Center for Integrative Genomics, University of Lausanne, Lausanne, Switzerland

**Keywords:** Biophysics, Cell biology

## Abstract

In eukaryotes, DNA is packed inside the cell nucleus in the form of chromatin, which consists of DNA, proteins such as histones, and RNA. Euchromatin, which is permissive for transcription, is spatially organized into transcriptionally inactive domains interspersed with pockets of transcriptional activity. While transcription and RNA have been implicated in euchromatin organization, it remains unclear how their interplay forms and maintains transcription pockets. Here we combine theory and experiment to analyze the dynamics of euchromatin organization as pluripotent zebrafish cells exit mitosis and begin transcription. We show that accumulation of RNA induces formation of transcription pockets which displace transcriptionally inactive chromatin. We propose that the accumulating RNA recruits RNA-binding proteins that together tend to separate from transcriptionally inactive euchromatin. Full phase separation is prevented because RNA remains tethered to transcribed euchromatin through RNA polymerases. Instead, smaller scale microphases emerge that do not grow further and form the typical pattern of euchromatin organization.

## Abstract (web summary)

How euchromatin organisation and transcription are related is unclear. Here, the authors observe the dynamics of euchromatin organization showing that accumulating RNA recruits RNA-binding proteins that together with transcribed euchromatin separate from non-transcribed euchromatin, forming microphases.

## Introduction

Chromatin is highly organized inside nuclei^1,2^. For example, transcriptionally repressed heterochromatin is often segregated from transcriptionally permissive euchromatin, giving rise to the B and A compartments, respectively^3,4^. Euchromatin itself is organized into transcriptionally inactive domains interspersed with pockets of transcriptional activity^2,5–7^. Considering that expressed genes are preferentially placed within pockets of transcriptional activity, these pockets together are also called the active nuclear compartment^1,8–14^. Although it has recently been shown that heterochromatin segregates from euchromatin via the physical process of phase separation^15–17^, it remains unclear how the interspersed pattern that is characteristic of the internal organization of euchromatin is established and maintained.

Physical mechanisms that could be applied to the organization of euchromatin have been proposed. It has been suggested, for example, that nuclear bodies, including those formed from RNA and RNA-binding proteins (RBPs), can displace transcriptionally inactive chromatin^18^. As the active nuclear compartment contains high levels of RNA and RNA-binding proteins^1,5^, this provides a potential mechanism for the selective exclusion of inactive chromatin from the active compartment. Moreover, transcribed genes often associate with assemblies of transcription factors, RNA polymerase II, and RNA transcripts, which may favor the selective retention of active chromatin within the active compartment^7,10,11,19–22^. The further observation that transcription pockets do not fuse with each other relates to an ongoing discussion on how chromatin domains that belong to the same compartment are prevented from fusing with one another^23–26^. Recent theoretical work has proposed polymer-based models where full phase separation is prevented^26–29^. A configuration where several phase domains persist and do not fuse, in these models, is described as microphase separation. Models based on microphase separation seem particularly well-suited to explain the internal organization of euchromatin, but their applicability to this organization has not been tested. Here, we combine experiments in pluripotent zebrafish cells and theory to show that euchromatin is organized in line with an active microemulsion model. Specifically, transcription sites establish pockets by displacing nontranscribed euchromatin and act as macromolecular amphiphiles that stabilize these pockets in a microphase-separated configuration.

## Results

### Transcription onset after mitosis organizes euchromatin

**Fig. 1:**
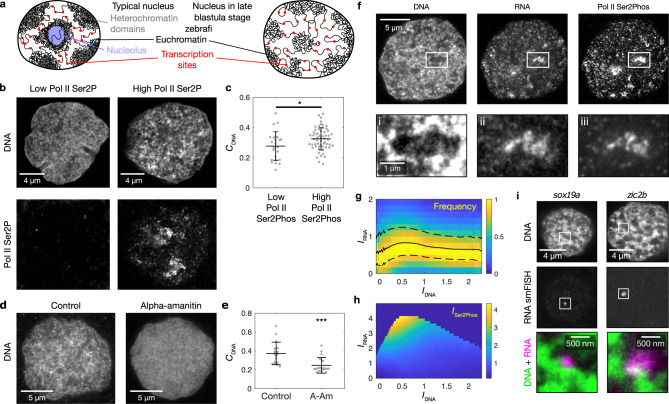
Transcription onset after mitosis establishes transcription pockets and euchromatin domains. **a** Sketch of nuclear compartmentalization in a typical nucleus and in the nucleus of a late blastula (sphere) stage zebrafish embryo. **b** Representative mid-sections of nuclei after mitosis and before transcription onset (Low Pol II Ser2Phos), and after transcription onset (High Pol II Ser2Phos). The same results were obtained in four independent experiments. **c** DNA image contrast (*C*_DNA_) for low and high Pol II Ser2Phos nuclei. Mean ± SD, * indicates *P* < 0.05 (*P* value 0.02 from a two-sided permutation test, *n* = 24,58 nuclei, from five different samples). **d** Representative nuclear mid-sections for control and α-amanitin treatment. The same results were obtained in two independent experiments. **e** *C*_DNA_ for control and α-amanitin (A-Am) treatment. Mean ± SD, *** indicates *P* < 0.001 (*P* value 0.00007 from a two-sided permutation test, *n* = 17,18 nuclei, from two different samples per condition). **f** Representative three-color micrographs showing DNA, RNA, and transcriptional activity (Pol II Ser2Phos) in a nuclear mid-section after transcription onset. The same results were obtained in four independent experiments. **g** 2D histogram displaying the frequency of observing a pixel with a specific RNA fluorescence intensity (*I*_RNA_) together with a given DNA intensity (*I*_DNA_). Solid and dashed lines are median and 25th and 75th percentile, respectively. *n* = 60 nuclei after transcription onset were used for analysis. **h** 3D pseudo-surface plot displaying the mean Pol II Ser2Phos intensity (*I*_Ser2Phos_) observed for a given *I*_RNA_ and *I*_DNA_. Same nuclei as in panel **g** were used for analysis. **i** Representative nuclear mid-sections showing RNA smFISH detection of transcription sites of the zygotic transcripts *sox19a* and *zic2b*. The same results were obtained in two independent experiments. All microscopy images in this figure were acquired by STED super-resolution microscopy.

To determine the role of transcription in euchromatin organization, we used zebrafish embryos at the late blastula (sphere) stage. In contrast to typical nuclei, zebrafish nuclei at this stage do not display heterochromatin^30,31^ or nucleoli^32,33^, allowing us to focus on euchromatin (Fig. 1a). Furthermore, cells divide approximately once per hour, which facilitates the frequent observation of transcription onset after mitosis and the concurrent establishment of euchromatin organization. To visualize DNA, RNA, and transcriptional activity within nuclei of intact cells, we dissociated embryos (Supplementary Fig. 1) and developed a protocol for three-color STED super-resolution microscopy (Supplementary Fig. 2). The DNA intensity profile inside nuclei is relatively smooth before transcription onset (Low Pol II Ser2Phos, elongating form of RNA polymerase II, in Fig. 1b and Supplementary Fig. 3). After transcription onset, a pattern of distinct DNA domains and DNA-depleted regions appears (High Pol II Ser2Phos in Fig. 1b and Supplementary Fig. 3). Indeed, image contrast, a measure that quantifies how strongly the intensity in different areas of the nucleus differs (see Supplementary Methods), is significantly increased after transcription onset, reflecting the observed formation of DNA domains (Fig. 1c). When we inhibited RNA polymerase II activity by injecting α-amanitin into zebrafish embryos, the distinct DNA domain pattern was not observed (Fig. 1d, e). These results indicate that RNA polymerase II-mediated transcription establishes a pattern of DNA domains.

### Transcription occurs in RNA-enriched domains

The role of transcription in the establishment of DNA domains could result from the accumulation of RNA, as well as from transcriptional activity itself. To dissect the individual contributions of RNA and transcriptional activity, we first investigated how they are spatially related to DNA domains in nuclei of transcriptionally active cells. We found that RNA is localized in regions that are generally depleted of DNA (Fig. 1fi, ii and Supplementary Fig. 4). Quantitative analysis confirmed that the highest intensities of RNA occur in regions of low DNA intensity (Fig. 1g). The RNA-binding proteins SC35 (canonical role in splicing) and hnRNPA1 (implicated in zebrafish development^34^) are found in the same regions (Supplementary Fig. 5). Inside these regions, low-intensity chromatin protrusions are retained (Fig. 1fi and Supplementary Fig. 4). It is specifically on these protrusions that we find peaks of transcriptional activity (visualized by Pol II Ser2Phos) (Fig. 1fiii and Supplementary Fig. 4), suggesting that the chromatin protrusions represent transcribed DNA. In support of this interpretation, a two-dimensional analysis that resolved transcriptional activity by DNA and RNA intensity revealed that the highest intensity of transcriptional activity is consistently found in locations with low DNA intensity and maximal RNA intensity (Fig. 1h). We confirmed that the transcription of RNA polymerase II-transcribed genes localizes to the interface of chromatin and RNA domains by visualizing nascent *sox19a* and *zic2b* transcripts by single-molecule fluorescence in situ hybridization (Fig. 1i). Together, our results suggest that transcription by RNA polymerase II results in distinct RNA-enriched regions that are segregated from domains of transcriptionally inactive chromatin. Transcriptionally active chromatin, as well as RNA-binding proteins, can be found in these RNA-rich regions.

### Euchromatin resembles an amphiphile-stabilized microemulsion

**Fig. 2:**
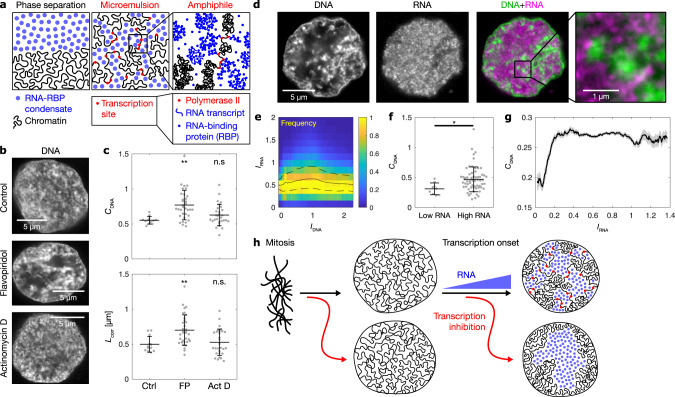
RNA accumulation establishes euchromatin domains, which are maintained in a finely dispersed pattern by transcriptional activity. **a** Cartoon representation of conventional phase separation and a microemulsion. Right panel focuses on the amphiphile in the microemulsion. **b** Representative micrographs of nuclear mid-sections obtained from cells treated for 30 min with the indicated inhibitors. The same results were obtained in two independent experiments. **c** Quantification of DNA image contrast (*C*_DNA_) and correlation length (*L*_corr_) for the different inhibitor treatments: control treatment (Ctrl), flavopiridol (FP), actinomycin D (Act D). Mean ± SD, ** indicates *P* < 0.01, n.s. indicates *P* ≥ 0.05 (*C*_DNA_ *P* values 0.002, 0.22 from a two-sided permutation test, *L*_corr_ *P* values 0.007, 1.2 from a two-sided permutation test, *P* values with Bonferroni correction for multiple testing, *n* = 12,34,29 nuclei from four, three, four different samples). **d** Representative nuclear mid-section of a flavopiridol-treated cell. The same results were obtained in three independent experiments. **e** 2D histogram displaying the frequency of pixel-level RNA intensity (*I*_RNA_) and DNA intensity (*I*_DNA_). Solid and dashed lines are median and 25th and 75th percentile, respectively. **f** Quantification of *C*_DNA_ and *L*_corr_ in flavopiridol-treated cells with low and high levels of RNA (the threshold for high RNA is *I*_RNA_ = 0.25). Mean ± SD, * indicates *P* < 0.05 (*P* value 0.03 from a two-sided permutation test, *n* = 9,62 from five different samples). **g** Running average (mean ± SEM), window width 0.1, windows with *n* ≥ 30 nuclei are drawn. **h** Sketch summarizing the experimental observations to this point. Microscopy data in **b**–**f** obtained by STED microscopy, **g** by spinning disk confocal microscopy.

These observations are reminiscent of a polymer solution in the process of phase separation. Indeed, known biomolecular condensates often involve multivalent protein/RNA interactions^35^, for example, in splicing speckles and nucleoli^36^. Our data suggest that RNA together with RNA-binding proteins segregates from euchromatin by phase separation, thereby forming distinct chromatin and RNA domains (Fig. 2a). In our case, however, phase separation is incomplete as domains remain small and do not coarsen into large domains. Small domain sizes could be the consequence of links between chromatin and the RNA-rich phase, via RNA polymerase II activity (Fig. 2a). This situation is a hallmark of systems undergoing microphase separation, which has recently been implicated in large-scale chromatin organization^23–29^. How microphase separation is achieved can be seen, for example, in microemulsions. Conventional microemulsions consist of two phases which tend to segregate, and an amphiphile that connects the two phases at the interface^37^. In these systems, phase separation leads to segregated microdomains while further growth of these domains is prevented by the amphiphile. The segregation of RNA from transcriptionally inactive chromatin, as seen in our experiments, suggests that RNA and transcriptionally inactive chromatin correspond to the two phases of a microemulsion. Following this logic, the tethering of transcripts to chromatin via RNA polymerase II at transcription sites would correspond to the amphiphile which has links to both.

### Chromatin-tethered RNA serves as a stabilizing amphiphile

If euchromatin is organized similar to such a microemulsion, it would be expected that the removal of the amphiphile destabilizes the microemulsion and results in coarsening of the domain pattern. To test this idea, we inhibited transcription with flavopiridol, a CDK9 inhibitor that results in the loss of transcribing RNA polymerase and its tethered transcripts from chromatin, while high overall levels of RNA are retained in the nucleus^38^ (Supplementary Fig. 6). As predicted, we observed that the pattern formed by DNA domains is markedly coarser in nuclei of inhibited cells when compared to nuclei of control cells (Fig. 2b). DNA domains are more pronounced, as reflected by an increased image contrast, and larger, as reflected by an increased correlation length, a measure that quantifies the length scale of patterns in the DNA intensity profile (Fig. 2c and Supplementary Fig. 7, for details, see Supplementary Methods). Importantly, RNA is localized to regions with low DNA intensity (Fig. 2d, e), as was observed in nontreated cells. Hence, the segregation into chromatin domains and RNA-enriched regions persists after the suppression of transcriptional activity. Flavopiridol-induced DNA domain coarsening disappeared after removal of the drug, suggesting that the observed changes are not due to toxic side effects of flavopiridol treatment (Supplementary Fig. 8). Together, these observations suggest that transcriptional activity is required to maintain RNA and chromatin domains in a finely interspersed pattern.

If transcriptional activity stabilizes the finely interspersed pattern of RNA and chromatin domains by establishing physical contacts between these domains, these contacts should be sufficient to maintain a finely interspersed domain pattern. To test this, we used the transcription inhibitor actinomycin D. In contrast to flavopiridol, which results in the loss of transcribing RNA polymerase from DNA^38^, actinomycin D arrests RNA polymerases during transcription^39^. The arrested polymerases are temporarily retained on DNA along with their associated transcripts^39^. Indeed, nascent mRNA transcription foci were retained on chromatin upon 30 min of actinomycin D treatment, while such foci disappeared upon flavopiridol treatment (Supplementary Fig. 9). As expected, no detectable coarsening of the DNA domains occurred upon actinomycin D treatment (Fig. 2b, c and Supplementary Fig. 10). These results imply that the physical contact between DNA and RNA that is established by RNA polymerase is required to maintain the interspersed pattern of DNA and RNA domains.

### RNA accumulation is required to form distinct phase domains

For microphase separation to occur, it is necessary that both phases are present. This suggests that the accumulation of RNA in the nucleus is required for the confinement of chromatin into distinct domains. After flavopiridol treatment, nuclei retained a range of RNA levels, due to differences in the amount of nuclear RNA at the time flavopiridol was applied. This allowed us to determine how different amounts of RNA in the nucleus contribute to euchromatin organization. As expected, we found that cells with low levels of RNA in the nucleus had lower DNA image contrast (Fig. 2f). When we recorded a large number of nuclei by spinning disk microscopy, we found that a relatively low level of RNA accumulation is sufficient to support the formation of distinct chromatin domains, and that DNA image contrast does not further increase with higher RNA levels (Fig. 2g). Together, this suggests that a minimum level of RNA needs to accumulate in the nucleus to establish a pattern of chromatin domains.

Our results to this point are summarized in Fig. 2h. In brief, transcription onset after mitosis establishes a finely interspersed pattern of mutually exclusive chromatin domains and transcription pockets. This interspersed pattern is maintained by DNA–RNA contacts established via RNA polymerases. As expected for a microemulsion^37^, the dissociation of these contacts results in coarsening of the pattern formed between the two phases. In nuclei not containing a significant amount of RNA, chromatin domains do not form.

### Simulations reproduce key features of euchromatin organization

**Fig. 3:**
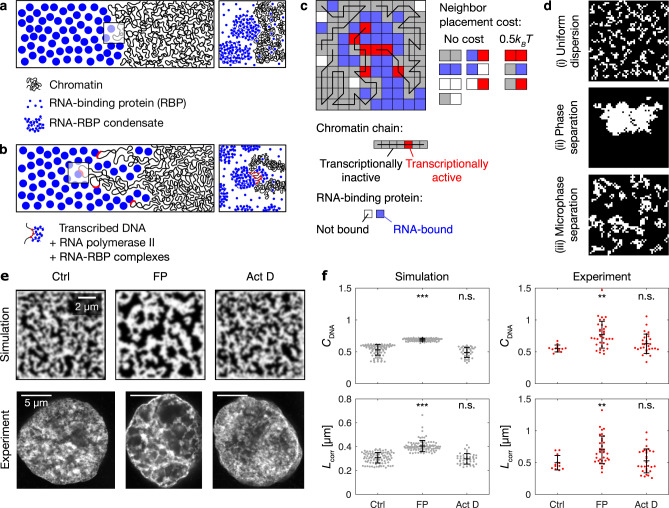
A microemulsion model reproduces key features of euchromatin organization. **a** Model mechanism 1: segregation of RNA-RBP condensates from chromatin. **b** Model mechanism 2: tethering of RNA-RBP complexes to transcriptionally active chromatin, forming amphiphilic connections. **c** Illustration of the lattice model for euchromatin organization, indicating chromatin chains and RNA-binding proteins with different states, as well as the costs for placing different types of lattice sites next to each other. See Supplementary Table 1 for model parameters. **d** Configurations of chromatin (white) in long-term simulations of the lattice model with (i) no RNA and no transcription, (ii) RNA and no transcription, and (iii) RNA and transcription. **e** Concentration profiles from simulations with example STED nuclear mid-sections from corresponding experimental conditions: control treatment (Ctrl), flavopiridol (FP), actinomycin D (Act D). The same results were obtained in four independent experiments. **f** Comparison of DNA image contrast (*C*_DNA_) and correlation length (*L*_corr_) from simulations and STED mid-nuclear sections. Mean ± SD, ** indicates *P* < 0.01, *** indicates *P* < 0.001, n.s. indicates *P* ≥ 0.05 (*C*_DNA_ *P* values <10^−5^, 0,33 from a two-sided permutation test, *L*_corr_ *P* values 0.003, 1.09 from a two-sided permutation test, *P* values with Bonferroni correction for multiple testing and resampling *n* matched to experiments, *n* = 96,93,46 simulations, for a description of the statistics of experiments, see Fig. 2c).

To investigate whether a microemulsion theory can explain the pattern of finely interspersed chromatin and RNA domains, we devised a simulation model with RNA-binding proteins and chromatin as the main components. We consider RNA-binding proteins (RBPs) to occur in two different states: unbound or bound to RNA, forming RNA–RBP condensates. Chromatin also occurs in two different states: transcriptionally active or transcriptionally inactive. Unbound RBPs mix with chromatin, whereas RNA–RBP condensates segregate from transcriptionally inactive chromatin (Fig. 3a). In addition, transcriptionally active chromatin has an affinity for RNA–RBP condensates. This captures the effect of tethering of RNA–RBP condensates to chromatin during the transcription process (Fig. 3b). Finally, RNA is produced by the transcription process, and accumulating RNA promotes the formation and growth of RNA–RBP condensates. Our system thus resembles a conventional microemulsion, consisting of two segregating phases and an amphiphile linking them. In our case, one phase is formed by a long polymers (chromatin). Moreover, the amphiphile in our model plays an active role: the RNA polymerase linking the two phases itself produces the RNA that is a key component of the RNA–RBP phase. Thus, we propose that the pattern of chromatin microphases can be understood as an active microemulsion.

We performed numerical simulations of this model using a two-dimensional lattice (Fig. 3c and “Methods”). Every lattice site can be occupied by one of four molecular species: transcriptionally inactive chromatin (gray), transcriptionally active chromatin (red), unbound RBPs (white), and RNA-bound RBPs (blue). Chromatin is represented by multiple linearly connected chains on the lattice that correspond to chromosomes (indicated by black lines). Individual sites of a given chromatin chain can switch from a transcriptionally inactive state to become transcriptionally active and produce RNA. RNA that accumulates at transcription sites converts unbound RBPs to RNA–RBP complexes, which condense and segregate from transcriptionally inactive chromatin. Beyond these changes of state, lattice sites undergo stochastic nearest neighbor swaps of their positions on the lattice which take into consideration neighbor-placement costs (Fig. 3c). The parameters describing reaction rates were taken from previous publications^38,40,41^.

To assess the different types of spatial organization that are possible in the model, we investigated the domain organization that emerges after a long time. First, we considered only transcriptionally inactive chromatin and RBPs. This resulted in a uniform dispersion of chromatin chains mixed with RBPs and no domain structure (Fig. 3di). Next, we executed simulations in which all chromatin was transcriptionally inactive and we considered all RBPs to be bound by RNA. Here, two distinct, clearly separated chromatin domains emerge, in line with a conventional phase separation scenario (Fig. 3dii). Lastly, we introduced transcriptionally active chromatin. In this case, an interspersed pattern of chromatin and RNA–RBP microdomains persists as a stochastic steady state of the system (Fig. 3ciii). As suggested by recent theoretical work^26–28^, ongoing switching of the transcriptionally active sites on chromatin influences the length scale of this microdomain pattern, but switching is not required for microphase separation (Supplementary Fig. 11). The simulations suggest that large-scale separation of RNA–RBP and chromatin domains can be prevented by transcription sites that play the role of amphiphiles in a microemulsion and maintain microdomains by linking the two phases. This supports our hypothesis that transcription sites (where transcripts are tethered to chromatin via RNA polymerase II) can act as linkers that maintain chromatin and RNA-enriched domains in a finely interspersed pattern.

### Simulations predict the effects of transcription inhibitors

To test whether this model can explain euchromatin organization in a concrete biological setting, we assessed how far it can reproduce our perturbation experiments in zebrafish cells. To this end, we extended the model by mimicking changes in transcription levels as they occur during the cell cycle (Supplementary Table 1). We then performed simulations addressing euchromatin organization in the absence or presence of transcriptional inhibitors. From these simulations, we determined chromatin concentration profiles that could be directly compared with STED microscopy images (Fig. 3e). We found that removing transcriptional linkers, but not just stopping transcription, results in an increase in contrast and correlation length in simulations, accounting for the experimental observations upon flavopiridol and actinomycin D treatment (Fig. 3f). Together, this confirms that the observed euchromatin organization can be explained by the interplay of two mechanisms: the segregation of RNA–RBP complexes from euchromatin, and the tethering of RNA–RBP complexes to transcriptionally active euchromatin via RNA polymerase II.

The proposed microphase separation scenario based on two very generic macromolecular mechanisms should be robust and also apply to other systems. We, therefore, assessed euchromatin organization in mouse embryonic stem cells. In contrast to zebrafish embryonic cells, nuclei of these cells do contain heterochromatin and nucleoli. Although this hampers image analysis, we found that, as in zebrafish blastomeres, flavopiridol but not actinomycin D results in euchromatin coarsening (increased correlation length, Supplementary Fig. 12). Moreover, euchromatin domains in the flavopiridol condition are more pronounced when RNA levels are higher (Supplementary Fig. 12). Thus, the core predictions of our theory are validated in a second cell type, without explicit consideration of the different molecular or cellular context.

### Transcription onset locally reorganizes euchromatin

**Fig. 4:**
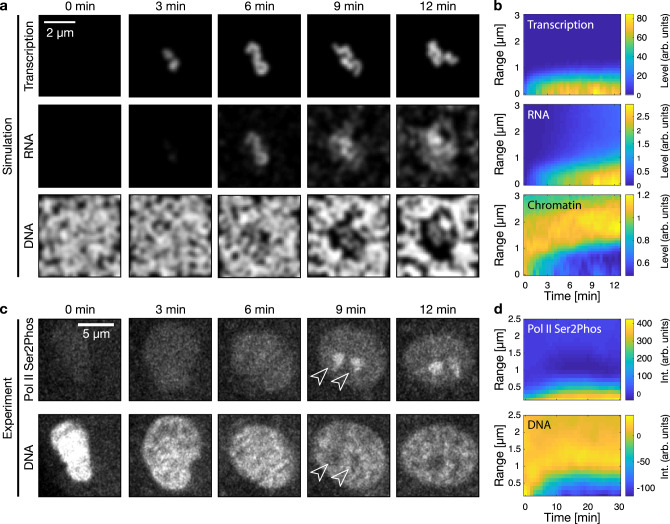
Transcription onset locally reorganizes euchromatin. **a** Representative simulated time-lapse of transcription onset (Transcription), RNA accumulation (RNA), and euchromatin organization (DNA) in the vicinity of a single transcriptionally activated chromatin chain. The same results were obtained in 12 simulations. **b** Radial analysis, starting at the time when transcription foci were first detected. The range indicates the radial distance from the centroid of a given transcription focus. Analysis averaged over 12 simulations. **c** Representative spinning disk confocal microscopy time lapse of elongating RNA polymerase II (Pol II Ser2Phos, visualized with phospho-specific antibody fragments) and DNA (SiR-DNA). Images show nuclear mid-sections of a single nucleus taken from zebrafish embryonic cell culture corresponding to the late blastula (sphere) stage. The same results were obtained in three independent experiments. **d** Radial analysis averaged over time-lapse recordings from 13 nuclei.

So far, we have focused on different states of euchromatin organization. To investigate the dynamics, we simulated the effects of transcription onset at an isolated transcription site (Fig. 4a). We found that the onset of transcriptional activity is followed by the accumulation of RNA–RBP complexes around the transcription site. A DNA-depleted region is established as the accumulating RNA–RBP complexes displace transcriptionally inactive chromatin, while transcriptionally active chromatin is retained within the chromatin-depleted, RNA-rich region. This sequence of events is conserved across repeated simulations (Fig. 4b). Next, to experimentally observe the dynamic process by which transcription organizes euchromatin in vivo, we followed two prominent transcription sites that precede nucleus-wide transcription in practically all nuclei of late blastula zebrafish embryos (Supplementary Fig. 13A). These foci localize to the microRNA miR-430 cluster (Supplementary Fig. 13B)^33,42,43^, which is highly transcribed in early embryonic development^41^. We used live cell-compatible antibody fragments to detect elongating RNA polymerase II^40,44,45^ in cultured embryonic cells in refractive index-matched medium^46^. We detected significant transcriptional activity at the two prominent foci within a few minutes after mitosis (Fig. 4c). At the sites where, and the time when we detected transcriptional activity, DNA was displaced (Fig. 4c). Microscopy of fixed cells confirmed that RNA accumulates with increasing transcriptional activity, and transcribed DNA is retained within the newly forming transcription pockets (Supplementary Fig. 14). As in the simulations, this sequence of events is conserved across different nuclei (Fig. 4d). Hence, in model simulations as well as in live cells, RNA accumulation in the vicinity of transcription sites displaces transcriptionally inactive chromatin while retaining transcriptionally active chromatin in the RNA-enriched region. Thus, our physical model can explain both how euchromatin organization is established, as well as how the resulting transcription pockets are maintained separately from each other.

## Discussion

We conclude that the formation of transcription pockets is driven by the accumulation of RNA–RBP complexes, which displace transcriptionally inactive euchromatin. We propose that the coalescence of these pockets into large-scale phase-separated domains is prevented by the tethering of RNA transcripts to transcriptionally active euchromatin via RNA polymerase II, resulting in a pattern of microphase domains. Euchromatin organization can be understood as a microemulsion, where RNA polymerase plays the role of an amphiphile linking segregated phases. Euchromatin is an active microemulsion because RNA polymerase synthesizes RNA transcripts in an active process. Interestingly, the activity of the amphiphile thus produces a key component of one of the phases involved.

Our observation that RNA and RNA-binding proteins drive the formation of transcription pockets is in line with previous work. It has long been known that RNA is enriched in the active compartment^1,2,5,7^, and the degradation of RNA results in the collapse of nuclear organization^47,48^. Nucleoli were shown to form by localized RNA production and, similar to transcription pockets, are strongly depleted of DNA^49^. Differently from transcription pockets, which are prevented from coarsening by anchored RNA transcripts, nucleoli are formed by the coalescence of several small droplets into a few large droplets within minutes after cell division^49^. Our model is agnostic to the identity of the RNA-binding proteins, but specific RNA-binding proteins with a role in euchromatin organization have previously been identified. SAF-A, for example, was identified as an RNA-binding protein that supports the transcription-dependent unfolding of euchromatin^50^. Interestingly, it was proposed that SAF-A/RNA complexes block attractive euchromatin–euchromatin interactions. In our lattice model, this SAF-A effect would be mathematically equivalent to the amphiphile effect, suggesting that SAF-A could be a molecular player underlying the microemulsion mechanism we propose.

Transcription has also been implicated in euchromatin organization before^1,2,5,7,10,11,32,50–54^. For example, transcription leads to the unfolding of transcribed chromatin regions, and transcribed genes have been shown to relocate to the active nuclear compartment^6,8,9,12,13^. Moreover, transcriptional activity has been shown to constrain the motion of chromatin domains^55,56^. This is in agreement with the model we propose, in which RNA connected to chromatin via elongating polymerase II anchors euchromatin in the active compartment. Transcription often takes place in macromolecular assemblies that bring together transcription factors, polymerases, transcribed genes, and RNA transcripts, also referred to as transcription factories^7,19,57–60^. It seems logical that such transcription factories could influence euchromatin organization. Indeed, recent super-resolution microscopy and genomic approaches have shown that sites with high transcription levels can associate into dynamic, polymerase II-enriched hubs that connect several transcribed genes in 3D space^10,11,20–22^. Transcription factories are most frequently described as structures with a diameter of 100 nm or less^7,19^. This is the approximate size of the transcription sites we find distributed throughout transcription pockets (Fig. 2a). Accordingly, it is possible that they constitute the amphiphiles proposed in our model.

Microphase separation stabilized by an amphiphilic linker provides a physical principle that clarifies how transcription and RNA accumulation together organize euchromatin. An increasing number of studies have successfully applied the physics of phase separation to nuclear organization^36,61,62^. Typically, conventional phase separation is invoked. One example of this is the formation of transcription factories. Here, both bridging-induced attraction (also called polymer–polymer phase separation) and liquid–liquid phase separation have been proposed as underlying mechanisms^20,25,63–69^. Both scenarios describe a mechanism by which transcribed genes could be anchored in RNA-enriched clusters, and are therefore compatible with the euchromatin organization as described in our model.

Another example is the separation of heterochromatin and euchromatin into distinct domains^15–17^. In this case, domains are established by liquid–liquid phase separation^15–17^. In conventional phase separation, domains tend to maximize their size. Here, we propose an important role for microphase separation, which can maintain domains of small scale^23–29^. Microphase separation has previously been proposed to play a role in the ordering of single chromosomes and specific genomic loci^29,70^. We now show how microphase separation, resulting from transcription and RNA accumulation can establish and maintain a finely dispersed pattern of transcription pockets that spans the whole nucleus. We propose that this versatile geometric pattern answers to two regulatory requirements: the segregation of euchromatin into an active and an inactive compartment, as well as a large interface between these compartments to facilitate access of transcription factors to activate target genes when needed.

## Methods

### Embryo dissociation and cell culture

Wild type zebrafish (TLAB) were maintained and raised under standard conditions. Embryos were obtained by natural mating. Embryos were dechorionated within 20 min of fertilization and kept at 28 °C. For dissociation into single cells, embryos in the late Oblong stage were immersed in 1 ml of deyolking buffer (10% v/v glycerol/H_2_O with 55 mM NaCl, 1.75 mMKCl, 1.25 mM NaHCO_3_) in low retention microcentrifuge tubes and vortexed at low speed until no intact embryo fragments could be observed. After centrifugation (1 min, 300 × *g*), the supernatant was aspirated and replaced with wash buffer (10% v/v glycerol/H_2_O with 110 mM NaCl, 3.5 mM KCl, 2.7 mM CaCl_2_, 10 mM Tris/Cl, pH 8.5), and tubes were vortexed at low speed to dissolve the cell pellet. After centrifugation (1 min, 300 × *g*), the supernatant was aspirated and replaced with 1 ml of PBS (all PBS in this study was Dulbecco’s formulation) with 0.8 mM CaCl_2_. Cells were cultured in this suspension for 30 min unless a different time is indicated. At the beginning of the time in suspension culture, tubes were briefly vortexed at low speed and then transferred into a rotator to prevent pellet formation.

### Single-molecule RNA fluorescence in situ hybridization

Fluorescence staining of fixed primary zebrafish cell cultures with Stellaris single-molecule fluorescence in situ hybridization (smFISH) probes was carried out with a protocol modified from our previous work on embryo sections^71–73^. Cultures were prepared, incubated, and fixed in the same way as for immunofluorescence staining. Fixed cell pellets were permeabilized with 70% ethanol at 4 °C for 1–2 h and subsequently washed (10% formamide and 2xSSC in nuclease-free water). Each tube containing a sample was incubated with 100 µl of probe mix (hybridization buffer with 0.5 µl of probe stock) overnight at 30 °C. Samples were then washed twice (wash buffer) at 30 °C and mounted in glycerol with DAPI (diluted 1:2500 from stock dilution of 1 mg/ml) or SiR-DNA (diluted 1:60 from stock dilution of 1 mM). The smFISH probes against the *zic2b*, *sox19b*, and *eif4g2a* transcripts were identical to those used by Stapel et al.^71–73^.

### Quantification of zygotic transcripts by RT-qPCR

The normalized concentrations of zygotic transcripts in whole embryos and primary zebrafish cell cultures were obtained by quantitative PCR from liquid nitrogen snap-frozen samples^74^. Primary zebrafish cell cultures were grown in ibidi imaging dishes, collected for RNA extraction into microcentrifuge tubes using hand-pulled glass pipette tips, and subsequently treated identically with samples containing whole embryos. RNA was extracted with the RNeasy Mini Kit (74104, Qiagen) and reverse-transcribed using the iScript cDNA Synthesis kit (1708891, BioRad Laboratories). The qPCR master mix contained SYBR green (AB-1158, Thermo Fisher Scientific) with Rox (R1371, Thermo Fisher Scientific; 100 nM) and primers at a final concentration of 500 nM. The primers used against the *vox*, *inka1a*, *mxtx2*, and *eif4g2a* transcripts were identical to those used by Joseph et al.^74^ and are listed in Supplementary Table 2.

### Transcription inhibition

#### α-amanitin

α-amanitin (A2263, Sigma) was dissolved and diluted to 0.2 mg/ml in H_2_O, and 1 nl of this solution was injected into embryos at the single-cell stage to deliver 0.2 ng of α-amanitin^75^. Control embryos were injected with 1 nl of H_2_O.

#### Flavopiridol

Flavopiridol (F3055, Sigma) was dissolved to 12.5 M (5 mg/ml) in DMSO, and diluted in PBS with 0.8 mM CaCl_2_ to a final concentration of 1 µM for the application in suspension cell culture. Control cell cultures were kept in PBS with 0.8 mM CaCl_2_, with the corresponding DMSO concentration.

#### Actinomycin D

Actinomycin D (A1410, Sigma) was dissolved to 1 mg/ml in DMSO, and diluted in PBS with 0.8 mM CaCl_2_ to final concentrations of 5 µg/ml for the application in suspension cell culture. Control cell cultures were kept in PBS with 0.8 mM CaCl_2_, with the corresponding DMSO concentration.

### Fixed sample microscopy

#### Preparation of fixed cells for fluorescence staining

To compact the cultured cells into a pellet, suspension cultures were centrifuged during the last minute of cell culture (300 × *g*). To fix the cells without perturbing the pellet, 8% formaldehyde in 1× PBS was added to the cell culture medium in a volume ratio of 1 in 4, to give an effective concentration of 2% formaldehyde. After 30 min of fixation at room temperature, tubes were centrifuged (1 min, 600 × *g*), and the supernatant aspirated. To increase the mechanical stability of cells, a secondary fixation step was carried out by applying 8% formaldehyde in PBS for 30 min at room temperature, followed by centrifugation (1 min, 800 × *g*) and aspiration. To permeabilize the cell membrane and the nuclear envelope, 0.5% Triton X-100 in PBS was applied for 10 min at room temperature, followed by three washes with PBS with 0.1% Tween-20 (PBST).

#### Immunofluorescence labeling

Immunofluorescence labeling started with blocking samples in 4% (w/v) BSA in PBST for 30 min at room temperature. Primary antibodies were diluted in 2% (w/v) BSA in PBST and left to incubate at 4 °C overnight. This was followed by three PBST washes at room temperature and subsequent application of fluorophore-conjugated secondary antibodies in the same way as the primary antibodies.

### Total zygotic RNA labeling

Total zygotic RNA was labeled using the Click-iT RNA labeling kit (C10330, ThermoFisher). 1 nl of 50 µM 5-ethynyl uridine (EU, diluted from 100 µM stock in H_2_O) was injected into the cytoplasm of the first cell following fertilization, so that transcripts produced following the one-cell stage would incorporate EU. Click labeling of incorporated EU with an Alexa-594 azide was carried out following the manufacturer instructions, applying 100 µl click labeling mix per microcentrifuge tube. When combined with immunofluorescence staining, click labeling was carried out after permeabilization and before BSA blocking.

### FISH labeling of primary miR-430 transcripts

miR-430 primary transcripts were labeled by RNA fluorescence in situ hybridization (RNA FISH) using a primary transcript probe kindly provided by Antonius van Boxtel^76^. FISH probes were in vitro transcribed from a linearized pGEMt_miR-430_ISH plasmid (NdeI restriction enzyme, New England BioLabs) using T7 polymerase. The in vitro transcription mix contained 2 µl of transcription buffer (Roche), 2 µl of DIG RNA labeling mix (Roche), 2 µl of DTT stock (0.1 mM stock concentration), 1 µl of RNAse inhibitor (Roche), 8 µl of linearized DNA, 4 µl of nuclease-free H_2_O, and 1 µl of T7 polymerase (produced in-house at Max Planck Institute of Molecular Cell Biology and Genetics) and was left to incubate at 37 °C for 2 h. In vitro transcription was followed by addition of 1 µl Turbo DNase (Ambion), incubation at 37 °C for 1 h, clean-up with RNeasy MinElute Cleanup kit (Qiagen), and dilution in hybridization buffer (500 ml formamide; 65 ml 20× SSC, pH 5,0; 10 ml EDTA 0.5 M; 50 mg Torula yeast; 2 ml of 10% Tween-20 (v/v); 5 ml of 20% SDS (manufacturer stock concentration); 2 ml of 50 mg/ml heparin stock, filled up to 1 l, aliquoted to 50 ml, and stored at −20 °C) to 50 mg/ml. The FISH procedure was started with one wash 50%/50% (v/v) methanol/PBST, followed by two washes with 100% methanol, then samples were placed at −20 °C overnight. After returning samples to room temperature, two washes in 50%/50% Methanol/PBST and two washes in PBST followed. 70 °C prewarmed hybridization buffer was added, and samples were incubated for 1 h at 70 °C. Samples were then incubated in prewarmed hybridization buffer with 1:25 hybridization probe for 4 h at 70 °C, followed by three washes in hybridization buffer (70 °C, 20 min each), one wash in 50%/50% (v/v) methanol/PBST (70 °C, 15 min exact), and three washes in PBST (room temperature, 10 min each), and 5% (v/v) blocking buffer (2% blocking reagent (Roche, 1,096,176) in 1× maleate buffer; maleate buffer: 150 mM maleic acid, 100 mM NaCl, pH 7.5, filter-sterilized, stored at room temperature) in PBST (room temperature, 20 min). Primary antibody incubation (mouse IgM monoclonal anti-Pol II Ser2Phos; anti-digoxigenin-POD, sheep Fab fragments) was in 2% BSA in PBST at 4 °C for 48 h, followed by three washes with PBST. FISH probes were revealed using the TSA Plus Cyanine 3 signal amplification kit (Perkin-Elmer), preparing 1 µl Cy3-Tyramide in 25 µl amplification buffer per sample, which was applied for 30 min at room temperature, followed by one wash in PBST. Incubation with secondary antibody (anti-mouse IgM-Alexa 488) was in 2% BSA in PBST at 4 °C overnight, followed by three washes in PBST.

### DNA labeling and mounting

DNA was labeled with DAPI or SiR-DNA (SC007, Spirochrome). DAPI was used for spinning disk confocal microscopy. DAPI was added directly into mounting media immediately before mounting at a concentration of 2 µg/ml. DAPI-stained samples were mounted in VectaShield H-1000, a non-setting liquid mounting medium. SiR-DNA was used for STED microscopy, RNA FISH labeled samples (FISH procedure induced high background on DAPI channel), and spinning disk confocal microscopy (equal or superior performance compared to DAPI). SiR-DNA staining produced no or very low signal in PBS, PBS + DABCO, or VectaShield H-1000, but the signal was extremely bright when samples were mounted in glycerol-rich media. For this reason, SiR-DNA-stained samples were mounted in glycerol. Because glycerol induced dissociation of several antibody combinations from the samples, immunofluorescence staining in these samples was followed by a postfixation step of 30 min in PBS with 4% formaldehyde, three washes in PBST, and a careful but thorough replacement of PBST with ~20 µl of pure glycerol. We then diluted the SiR-DNA stock (1 mM in DMSO) in glycerol of which we spiked 1 µl into every sample immediately before mounting. The dilution of SiR-DNA in glycerol was adjusted so that upon addition to the 20 µl mounting medium the desired dilution was reached (1:60 in all cases except after α-amanitin treatment, where 1:400 was used; all dilutions produced sufficient signal). Samples were mounted by spotting of mounting medium with resuspended cells onto regular microscope slides, applying #1.5 coverslips, and sealing with nail polish.

### STED super-resolution microscopy of fixed cells

Measurements were performed on a commercial confocal STED microscope (Abberior Instruments, Göttingen, Germany) with pulsed laser excitation (490 nm, 560 nm, 640 nm, 40 MHz), beam-scanning module (line frequency 3 kHz), a pulsed STED laser (775 nm, 40 MHz, spatial light modulator to produce the donut) and single-photon counting APD detectors. Multicolor STED imaging with the single 775-nm STED laser was done by using chromatic separation of the fluorophores in combination with line-interleaved (time) excitation and detection. For the 560-nm and 640-nm channels, we used the dyes Alexa 594 and SiR, respectively. For the 490-nm channel, we used the long Stokes shift dye Abberior STAR 470 SXP, which emits in the 560-nm and 640-nm channel and can be effectively depleted by the 775-nm STED laser. To account for direct excitation of the SiR dye by the STED laser, we recorded the 640-nm channel additionally with only the STED laser-activated. This channel was then subtracted from the SiR 640-nm channel.

### Spinning disk confocal microscopy of fixed cells

Fixed cells were imaged using the Andor Revolution platform with Borealis extension, equipped with an Olympus silicone oil immersion objective (UPLSAPO 100XS, NA 1.35), recording with a single iXon Ultra 888 EMCCD camera. Acquisition settings were kept consistent across the different samples of a given experiment.

### Light-sheet imaging of whole fixed embryos

Fixed whole embryos were prepared, fluorescently stained, and imaged using a Zeiss Z1 light-sheet microscope exactly as described by us in a previous publication (Joseph et al.^74^). Pol II Ser2Phos was labeled by immunofluorescence, using mouse IgM anti-Pol II Ser2Phos primary antibody (H5, 1:500) and anti-mouse IgM secondary antibody (conjugated with Alexa 488, dilution 1:1000). DNA was stained by adding 1 µg/ml DAPI during secondary antibody incubation.

### List of antibodies

#### Primary antibodies

- Mouse IgM anti-Pol II CTD Ser2Phos (H5), monoclonal, ab24758 Abcam
- Dilution: 1:500 for light-sheet microscopy
- Rabbit IgG anti-Pol II CTD Ser2Phos, monoclonal, ab193468 Abcam
- Dilutions: 1:200 for STED microscopy, 1:1000 for confocal microscopy
- Rat IgG anti-H3 Ser28Phos, monoclonal, ab10543, Abcam
- Dilution: 1:1000 for confocal microscopy
- Sheep IgG anti-digoxigenin Fab fragments, conjugated with horseradish peroxidase, 1207733 Roche; dilution: 1:500 for fluorescence in situ hybridization
- Mouse IgG anti-SC35, monoclonal, 556363 BD Biosciences
- Dilution 1:100 for confocal microscopy
- Mouse IgG anti-hnRNPA1, monoclonal CA1, concentrated cell supernatant; a kind gift from the Black Laboratory
- Dilution 1:500 for confocal microscopy

#### Secondary antibodies

- Goat anti-mouse IgM, conjugated with Alexa 488, A21042 Thermo Fisher
- Dilution: 1:1000 for light-sheet microscopy
- Goat anti-rabbit IgG, conjugated with STAR 470 SXP, 2-0012-008-9, Abberior
- Dilution: 1:200 for STED microscopy
- Donkey anti-rabbit IgG, conjugated with Alexa 488, A21206 Thermo Fisher
- Dilution 1:1000 for confocal microscopy
- Donkey anti-rat IgG, conjugated with Alexa 488, A21208 Thermo Fisher
- Dilution 1:1000 for confocal microscopy
- Goat anti-rat IgG, conjugated with Alexa 647, A21247 Thermo Fisher
- Dilution 1:1000 for confocal microscopy

### Live cell microscopy

#### Preparation of antibody fragments for use in live-cell microscopy

Fluorescently labeled antibody fragments (Fabs) specific to Pol II Ser5Phos and Pol II Ser2Phos were prepared from monoclonal antibodies specific to Pol II Ser5 and Ser2 phosphorylation^40,44,77^. Monoclonal antibodies were digested with Ficin (ThermoFisher Scientific), and Fabs were purified through protein A-Sepharose columns (GE Healthcare) to remove Fc and undigested IgG. After passing through desalting columns (PD MiniTrap G25; GE Healthcare) to substitute the buffer with PBS, Fabs were concentrated up to >1 mg/ml using 10 k cut-off filters (Amicon Ultra-0.5 10 k; Merck), Fabs were conjugated with Alexa Fluor 488 (Sulfodichlorophenol Ester; ThermoFisher Scientific) or Cy3 (N-hydroxysuccinimide ester monoreactive dye; GE Healthcare) to yield ~1:1 dye:protein ratio. After the buffer substitution with PBS, the concentration was adjusted to ~1 mg/ml.

#### Preparation of live cells for fluorescence microscopy

Directly the following fertilization, zebrafish embryos were pronase-dechorionated and 1 nl of a mix made up of 0.3 µl Alexa 488-conjugated Pol II Ser5Phos Fab, 1.7 µl Cy3-conjugated Pol II Ser2Phos Fab, 0.2 µl 1 mM SiR-DNA, and 0.1 µl 10× Phenol Red was injected into the cytoplasm at the single-cell stage. Embryos were grown at 28 °C and dissociated into single cells at the High stage. These cells were mounted in D-PBS supplemented with 0.8 mM CaCl_2_, 0.7% UltraPureTM (Thermo Fisher Scientific, Cat. No. 16520050), and iodixanol (adjusted to match a refractive index of 1.3615) in an ibidi glass-bottom dish^46^. During the time required to mount the cells and start microscopy, cells had undergone one to two divisions. In intact embryos, cells also undergo one or two cell divisions during the developmental progression from the High to Oblong or Sphere stage. Thus, we acquired live microscopy images from cultured cells that should most closely correspond to cells at the Oblong or Sphere stage in the intact embryo.

#### Confocal microscopy of live cells

Live cell cultures were imaged using the Andor Revolution platform with Borealis extension, equipped with an Olympus silicone oil-immersion objective (UPLSAPO 100XS, NA 1.35), recording with a single iXon Ultra 888 EMCCD camera. Image data were acquired for up to four cell clones in parallel. A full three-color z-stack could be obtained every minute for all cell clones. Time-lapses were recorded over periods of up to 90 min, during which cells continuously displayed cell divisions, suggesting no obvious phototoxicity.

### Suspension culture of mouse embryonic stem cells

Mouse embryonic stem cells (R1/E murine ES-cells, subclones from R1 originals (Toronto), derived from crossing 129×1/SvJ and 129S1/SV- + ^p^ + ^Tyr-c^ Kitl ^Sl-J^/ + ) were expanded in suspension culture in media supplemented with 1i/LIF, using a simplified protocol^78^. Cell cultures were distributed in a 24-well plate, dosed with 1 mM EU for 4 h, and subsequently treated with transcription inhibitors (spiked in) for 30 min. Incubation was at 37 °C and CO_2_-controlled atmosphere. Cells were transferred to low-retention microcentrifuge tubes and fixed by 1:3 (v/v) addition of 8% formaldehyde in PBS with 0.8 mM CaCl_2_ with spin-down at 800 × *g*. Further treatment for immunofluorescence, RNA click labeling, and DNA staining was identical to that of primary zebrafish cell cultures obtained from zebrafish embryos.

### Image preparation and analysis

#### Software used for image preparation and analysis

Microscopy image preparation was done using FIJI^79^ and MatLab, the latter relying on the Open Microscopy Environment plugin for image import^80^. Further data processing was carried out in MatLab. The resulting figures were prepared for publication using MatLab and Adobe Illustrator.

#### Segmentation of nuclei

The nuclei in STED images are segmented by applying Otsu’s method for adaptive thresholding to the DNA channel. In some cases, the resulting segmentation mask contains holes, which are removed by a filling step. Distortion and artifacts from out-of-focus light are seen at the boundaries of nuclei. To remove these imaging imperfections from further structural analysis, the segmentation masks are eroded before further analysis.

Spinning disk confocal microscopy data contain several nuclei and consist of a stack of multiple images in the z direction. An initial segmentation step based on a fixed, manually chosen threshold is applied to the DNA channel to obtain substacks containing individual nuclei. To extract a single image close to the middle of the nucleus in a given stack, the z section with the highest mean intensity in the DNA channel is selected for further analysis. In this image, the nucleus is segmented using the same approach as described for STED images above. Images from STED and spinning disk confocal microscopy can be analyzed in the same manner from here on.

#### Calculation of nuclear intensities

The mean nuclear intensity of a given color channel is extracted using the nuclear segmentation masks obtained from the DNA channel. These mean nuclear intensities contain contributions from the actual nuclear signal and also image background intensity. To remove image background intensity, the fluorescence in the cytoplasm is determined and subtracted from the total nuclear intensity. The cytoplasmic intensity is determined using a segmentation shell that is created by an outward dilation of the nuclear segmentation mask^74,81^.

#### Calculation of image contrast

The DNA image contrast (*C*_DNA_) is calculated as the root-mean-square contrast of the individual pixels’ intensities (*I*_*n*_) and normalized by the mean intensity, $\widehat I$,

$$C_{{\mathrm{DNA}}} = \frac{1}{{\widehat I}}\sqrt {\frac{1}{{N - 1}}\mathop {\sum}\nolimits_{n = 1}^N {\left( {I_n - \widehat I} \right)^2} } = \frac{{\sigma _I}}{{\widehat I}},$$

where *σ*_I_ is the standard deviation. This is equivalent to the coefficient of variation of *I*_*n*_.

The *C*_DNA_ of samples prepared, stained, and imaged under comparable conditions and identical settings can be quantitatively compared. To compare images obtained under different conditions, background intensity correction is required. This is also required when comparing microscopy images and simulated chromatin concentration profiles. An appropriate background correction can be calculated assuming an offset to the individual intensity values,

$$I_n^\prime = I_n + I_{{\mathrm{offset}}}.$$

This leads to a changed image contrast value,

$$C_{{\mathrm{DNA}}}^\prime = \frac{{\sigma _I}}{{\widehat I + I_{{\mathrm{offset}}}}} = aC_{{\mathrm{DNA}}},a = \frac{{\widehat I}}{{\widehat I + I_{{\mathrm{offset}}}}}.$$

Thus, by obtaining *I*_offset_, the background intensity in the DNA channel from regions outside the nucleus, *C*_DNA_ values obtained under different conditions can be corrected.

#### Calculation of correlation length

The correlation length of the DNA intensity distribution (*L*_corr_) is determined in two main steps. First, the radial correlation function, *g*(*r*), is extracted. We use a definition of the radial correlation function that takes into consideration the segmentation mask covering the inside of the cell nucleus. Considering a DNA pixel intensity image *I*_(*i,j*)_, with the two-dimensional position of the pixel indicated by *i* and *j*, and an associated segmentation mask $\sigma _{(i,j)} = \{ 0,1\}$, the radial correlation function at a distance *r* is

$$g_x\left( r \right) = \left[ {\mathop {\sum}\limits_{i = 1,j = 1}^{N_x - r,N_y} {\left( {\sigma _{i,j}I_{i,j}\sigma _{i,j + r/l}I_{i,j + r/l}} \right)} } \right]/\left[ {\mathop {\sum}\limits_{i = 1,j = 1}^{N_x - r,N_y} {\sigma _{i,j}\sigma _{i,j + r/l}} } \right]$$

in the case of shifting in the *x* direction. Note that, due to the pixel resolution *l*, *g*_*x*_(*r*) is only evaluated at discrete intervals $r = 0,l,2l, \ldots ,N_xl$. The equivalent calculation is carried out for shifts in *y* direction to obtain *g*_*y*_(*r*). The combined radial correlation function then is $g(r) = (g_x + g_y)/2$. Before the calculation of *g*(*r*) the intensities of all color channels are normalized by the respective color channels’ mean intensity in the segmented nucleus, followed by subtraction of the mean intensity in the segmented nucleus.

Second, to obtain *L*_corr_, an exponential decay function is fitted to *g*(*r*). To this end, the function

$$f\left( {r\left| {L_{{\mathrm{corr}}}} \right.} \right) = g_\infty + (g_0 - g_\infty )e^{ - r/L_{{\mathrm{corr}}}}$$

is adjusted to *g*(*r*) by optimization of the value of *L*_corr_. Here, $g_0 = g(r = 0)$ and *g*_∞_, representing the plateau level of the decaying correlation function, was approximated by the mean value of *g*(*r*) in the interval of *r* from 2.0 to 3.5 μm.

A common approach to structural characterization, Fourier analysis, cannot be used. Given that the structural analysis has to be contained to the inside of cell nuclei, domains with irregular boundaries need to be analyzed. It is not clear how Fourier analysis can be applied to such irregular domains in a straight-forward manner.

#### Intensity distributions of one-color channel with respect to another color channel

To determine the relationship between the intensity profiles of different color channels, we analyze the distribution of fluorescence intensities of a given channel (A) with respect to intensities in another color channel (B). To this end, all pixels of an image are binned based on the intensities of channel B. Then, the mean intensity on channel A of all pixels within a given bin is calculated. This analysis reveals the intensity distribution of color channel A with respect to intensities in the color channel B.

The same principle can be applied to resolve a color channel A by the intensities of two other color channels, B and C. Instead of binning pixels only with respect to a single color channel (one-dimensional binning), the pixels are now binned with respect to two color channels (two-dimensional binning).

#### Analysis of live cell images

At every time point, nuclei are segmented based on Pol II Ser5Phos Fab signal. Specifically, we first use the fact that the signal of Pol II Ser5Phos Fab occurred in nuclei but also throughout the cytoplasm to segment cells from the background using an Otsu threshold. Second, we use the higher signal intensity within nuclei to segment nuclei from the cytoplasm, by applying an Otsu threshold within the segmented cells. When the Otsu metric is below 0.65, nuclei are segmented. Otherwise, it is assumed that the Fab pool was released to the cytoplasm due to nuclear envelope breakdown during mitosis, and no nuclei are segmented. For all pixels within segmented nuclei, their 3D distance to the nearest non-segmented pixel is calculated. To segregate nuclei that are too close to be directly segmented, a watershed segmentation is initiated from the maxima of this distance map. The segmented nuclei are first automatically tracked through time by their centroid distance. Where tracks have gaps or are not correctly connected, tracks are then manually corrected.

To analyze spatial organization around the two prominent transcription sites, we carried out a radial intensity analysis that is centered on these. Before any analysis, all fluorescence images are locally corrected for background intensity: each *xy* image is copied, filtered with a Gaussian kernel (kernel width of $\sigma = 2.38\,\upmu {\mathrm{m}}$), and subtracted from the unprocessed image. Transcription sites are segmented with an Otsu threshold applied to the Pol II Ser2Phos channel, and the two largest objects are retained, assuming that they are the two prominent transcription sites. For both these objects, the centroid is determined, and the *xy* section containing the centroid is extracted for radial analysis. Within these *xy* sections, the pixel containing the centroid is marked as the starting point of the analysis. With respect to the radial range of the analysis, this pixel is located at a range of 0, referring to the center of the transcription site. The first radial outward step now marks all eight neighbors of this initial pixel and refers to a radial range of one pixel. A radial range of two pixels is reached by marking the next line of outward-lying neighbors, and so forth for all further ranges. At all ranges, the mean intensities of Fab Pol II Ser5Phos and SiR-DNA signal within the pixels belonging to this radial range are calculated. This procedure produces an intensity curve for all color channels at different radial ranges with respect to the centroid of a given transcription site. To average over the transcription sites of several nuclei, the tracked nuclei were temporally aligned by the first time at which two transcription foci could be detected in a given nucleus. Two-dimensional images of intensity resolved by radial range and time were then created for each tracked nucleus. These were averaged over all tracked nuclei to create final plots.

### Lattice model

The chemical reactions included in our lattice model only locally inter-convert species so that this part of the model is evaluated independently of spatial rearrangements. Specifically, a fixed time interval Δ*t*_chem_ is chosen, and every time this interval has elapsed, all cells are addressed for the occurrence of chemical conversion. For each particle, there is always only one possible chemical conversion, with a given rate *k*. Thus, conversions are actually executed with a probability $P_{{\mathrm{chem}}} = \Delta t_{{\mathrm{chem}}} \cdot k$. Accordingly, we have chosen $\Delta t_{{\mathrm{chem}}} = 0.1/k_{{\mathrm{max}}}$, where $k_{{\mathrm{max}}}$ is the fastest conversion rate in the model. The conversion reactions that affect single species are

• Transcriptionally inactive chromatin into transcriptionally active chromatin ($k_{{\mathrm{chrom}}}^ +$)

• Transcriptionally active chromatin into transcriptionally inactive chromatin ($k_{{\mathrm{chrom}}}^ -$)

• Production of an RNA transcript at a transcriptionally active chromatin site ($k_{{\mathrm{RNA}}}^ +$)

• Transfer of an RNA transcript from a chromatin site to a neighboring RBP site ($k_{{\mathrm{RNA}}}^{{\mathrm{transfer}}}$)

• Spontaneous decay of an RNA transcript ($k_{{\mathrm{RNA}}}^ -$)

In addition, to mimic the process of different genes turning on and off, as is seen in the real zebrafish embryo cells, we introduce a switching of chains between a permissive and a restrictive state. Switching of chromatin chains between a permissive and restrictive state is carried out during the chemical reaction step. Chains switch into the permissive state with a rate $k_{{\mathrm{chain}}}^{{\mathrm{on}}}$, and into the restrictive state with a rate $k_{{\mathrm{chain}}}^{{\mathrm{off}}}$. Chromatin sites whose containing chain is in the permissive state can become transcriptionally active with the according rate $k_{{\mathrm{chrom}}}^ +$, but sites on a currently restrictive chain cannot become transcriptionally active. Any transcriptionally active site can always become transcriptionally inactive, irrespective of the state of the chain it belongs to.

The cells on the lattice are spatially rearranged by the proposal and probabilistic acceptance of direct neighbor swaps. For every iteration step, two direct neighbors are randomly chosen, and the swap is executed with a probability $P_{{\mathrm{swap}}} = exp(\Delta E_{{\min} -\Delta E}),$ where Δ*E* is the energy difference resulting from a proposed swap operation, and Δ*E*_min_ the largest possible drop in energy possible by any swap operation. Δ*E* is calculated based on the energy level *E*_pre_ before the swap operation and the energy level E_post_ after the swap operation, $\Delta E = E_{{\mathrm{post}}} - E_{{\mathrm{pre}}}$. The energy levels are calculated based on direct neighborhood relationships, where mismatching neighbors are assigned an energetic cost *w* > 0 (stated in units of thermal energy, $k_BT$). The value of *E* for a given configuration is obtained simply by summing over all mismatching neighbors. The species combinations for which a direct neighbor relationship gives an energetic penalty are transcriptionally inactive chromatin/RNA-bound RBP (RBP with one or more RNA transcripts), transcriptionally inactive chromatin/transcriptionally active chromatin, and transcriptionally active chromatin/transcriptionally active chromatin. All neighborhood relationships include diagonal neighbors (8-neighborhood). In the case that a swap operation is executed, the RNA transcripts located at a given lattice site are swapped to the new position as well, representing the binding of RNA to transcriptionally active chromatin or RBPs.

Different initial conditions and time-dependent changes in parameter values were applied to represent different situations that should be simulated. In the simulations that were carried out to explore the possible phenomena resulting from our model, chains were placed in 100 boxes of size 5 × 5 lattice sites each, in a lattice of the overall size of 50 × 50 lattice sites. In each cell, a chain of five sites length was placed. In the “Uniform Dispersion” case, no chemical reactions were executed during the simulation, so that only transcriptionally inactive chromatin and unbound RBP was present. In the “Phase Separation” case, the simulation was prepared to contain only chromatin and all RBP were bound by RNA, RNA was prevented from decaying. In the “Microphase Separation” case, all chromatin chains were turned permissive to reduce model complexity, and individual sites became transcriptionally active and transcriptionally inactive as explained above. In all three cases, chromatin did not attach to the lattice boundary, and the lattice was padded with RNA–RBP complexes around the boundary.

In the simulations that should approximate the changes in chromatin organization following cell division, all chromatin chains were initialized as permissive for transcription. Chromatin chains of length 50 were densely packed within boxes of 10 × 10 lattice sites, with an overall number of 100 chains on a 100 × 100 lattice. For an initial phase of 10 min, all chemical reaction rates were maintained at 0, to allow relaxation of the chromatin chains. After 10 min, the nonzero parameter values were applied. To implement the application of transcriptional inhibitors, parameter changes were applied during a running simulation, and the simulations were continued for a simulated time of 30 min. Specifically, in the case of flavopiridol inhibition, chromatin could not become transcriptionally active ($k_{{\mathrm{chrom}}}^ + = 0$), all other parameters remained unchanged. In the case of actinomycin D, chromatin could not become newly transcriptionally active ($k_{{\mathrm{chrom}}}^ + = 0$), but also remained in the transcriptionally active state to mimic amphiphile retention ($k_{{\mathrm{chrom}}}^ - = 0$), and RNA production and RNA transfer off of chromatin were blocked ($k_{{\mathrm{RNA}}}^{ + = 0},k_{{\mathrm{RNA}}}^{{\mathrm{transfer}}} = 0$), as well as RNA decay specifically for RNA on chromatin ($k_{{\mathrm{RNA}}}^ - = 0$ only on chromatin sites). Chromatin that touched the boundary layer got permanently attached to the margin, and the lattice was padded with inactive chromatin.

In the simulations that should approximate transcription onset at the miR-430 locus, 5 × 5 chains were initiated in a 50 × 50 lattice. Only the chain in the center was turned permissive for transcription, after an initial relaxation time of 10 min. In these simulations, chromatin that touched the boundary layer was not permanently tethered, but the lattice was again padded with inactive chromatin to provide neighbors for interaction.

The simulation code consists of a core library in C++ and several utility functions (bash and python) to interact with this core library on local computers as well as the bwUniCluster at Karlsruhe Institute of Technology. All code is available as open-source and documented at https://github.com/lhilbert/active-microemulsion.

In those cases where simulation outputs were compared to experimental intensity distributions, the according species’ lattice distributions were translated into smoothed concentration profiles. Specifically, a pixel size of 100 nm was assumed, and a Gaussian blur filter with *σ* = 120 nm (to compare with STED micrographs), *σ* = 450 nm (to compare with spinning disk micrographs), or *σ* = 150 nm (to compare with live-cell micrographs). The concentration profile to compare with DNA intensity distributions was produced from chromatin in the simulation, the concentration profile to compare with RNA intensity was produced from RNA particles, the concentration profile to compare with Pol II Ser2Phos intensity was produced from transcriptionally active chromatin.

### Statistical testing: permutation tests with multiple comparison correction

The statistical hypothesis testing is based on permutation tests. To enable the comparison of *P* values against the common significance levels of single comparison tests (n.s. for *P* ≥ 0.05, * for *P* < 0.05,** for *P* < 0.01, *** for *P* < 0.001), the Bonferroni correction was applied to the final *P* values as a prefactor. In some cases, this results in *P* values that are larger than 1 due to the correction factor, which are to be interpreted as a n.s. conclusion. The computational resampling procedure was carried out to a *P* value accuracy of 10^−5^, smaller *P* values are stated as *P*  < 10^−5^.

### Reporting summary

Further information on research design is available in the Nature Research Reporting Summary linked to this article.

## Supplementary information

Supplementary Information

Reporting Summary

## Data Availability

The authors declare that all data supporting the findings of this study are available within the article and its supplementary information files or from the corresponding author upon reasonable request.

## Code availability

All simulation code is available as a GitHub open source repository (10.5281/zenodo.4424642)^82^.
