# Author Correction: Transcription organizes euchromatin via microphase separation

**DOI:** 10.1038/s41467-021-24517-7

**Published:** 2021-07-06

**Authors:** Lennart Hilbert, Yuko Sato, Ksenia Kuznetsova, Tommaso Bianucci, Hiroshi Kimura, Frank Jülicher, Alf Honigmann, Vasily Zaburdaev, Nadine L. Vastenhouw

**Affiliations:** 1 Center for Systems Biology Dresden, Dresden, Germany; 2 Max Planck Institute of Molecular Cell Biology and Genetics, Dresden, Germany; 3 Max Planck Institute for the Physics of Complex Systems, Dresden, Germany; 4 Tokyo Institute of Technology, Yokohama, Kanagawa Japan; 5 Center for Advancing Electronics Dresden, Technical University Dresden, Dresden, Germany; 6 Cluster of Excellence Physics of Life, Technical University Dresden, Dresden, Germany; 7 Present Address: Institute of Biological and Chemical Systems, Karlsruhe Institute of Technology, Eggenstein-Leopoldshafen, Germany; 8 Present Address: Zoological Institute, Karlsruhe Institute of Technology, Karlsruhe, Germany; 9 Present Address: Friedrich-Alexander Universität Erlangen-Nuremberg, Max Planck Zentrum für Physik und Medizin, Erlangen, Germany; 10 Present Address: Center for Integrative Genomics, University of Lausanne, Lausanne, Switzerland

**Keywords:** Biophysics, Cell biology

Correction to: *Nature Communications* 10.1038/s41467-021-21589-3, published online 1 March 2021.

In the original version of this Article, the text in the Methods section describing the lattice model algorithm incorrectly stated “For every iteration step, two direct neighbors are randomly chosen, and the swap is executed with a probability *P*_swap_ = *esp*(−Δ*E*/Δ*E*_min_)”. This error has now been fixed in the PDF and HTML version on the Article to read “For every iteration step, two direct neighbors are randomly chosen, and the swap is executed with a probability *P*_swap_ = *exp*(Δ*E*_min−Δ*E*_).”.

