# Supplementary material for: Transcription organizes euchromatin via microphase separation
Source: Nat Commun. 2021 Mar 1;12:1360. doi: 10.1038/s41467-021-21589-3 (PMC7921102; doi:10.1038/s41467-021-21589-3)
Supplement: Supplementary file 1 — Supplementary Information [file 41467_2021_21589_MOESM1_ESM.pdf]

Supplementary Materials

Transcription organizes euchromatin via microphase separation

Lennart Hilbert[1,2,3,7,8], Yuko Sato[4,11], Ksenia Kuznetsova[2,11], Tommaso Bianucci[2,3,11], Hiroshi Kimura[4], Frank Jülicher[1,3,5,6], Alf Honigmann[2], Vasily Zaburdaev[1,3,9,12] & Nadine L. Vastenhouw[2,10,12,@]

[1]Center for Systems Biology Dresden, Dresden, Germany. [2]Max Planck Institute of Molecular Cell Biology and Genetics, Dresden, Germany. [3]Max Planck Institute for the Physics of Complex Systems, Dresden, Germany. [4]Tokyo Institute of Technology, Yokohama, Kanagawa, Japan. [5]Center for Advancing Electronics Dresden, Technical University Dresden, Dresden. [6]Cluster of Excellence Physics of Life, Technical University Dresden, Dresden, Germany. [7]Present address: Institute of Biological and Chemical Systems, Karlsruhe Institute of Technology, Eggenstein-Leopoldshafen, Germany. [8]Present address: Zoological Institute, Karlsruhe Institute of Technology, Karlsruhe, Germany. [9]Present address: Friedrich-Alexander Universität Erlangen-Nuremberg, Max Planck Zentrum für Physik und Medizin, Erlangen, Germany; [10]Present address: Center for Integrative Genomics, University of Lausanne, Lausanne, Switzerland. [11]These authors contributed equally: Yuko Sato, Ksenia Kuznetsova, Tommaso Bianucci. [12]These authors jointly supervised this work: Vasily Zaburdaev, Nadine L. Vastenhouw. [@]email: nadine.vastenhouw@unil.ch

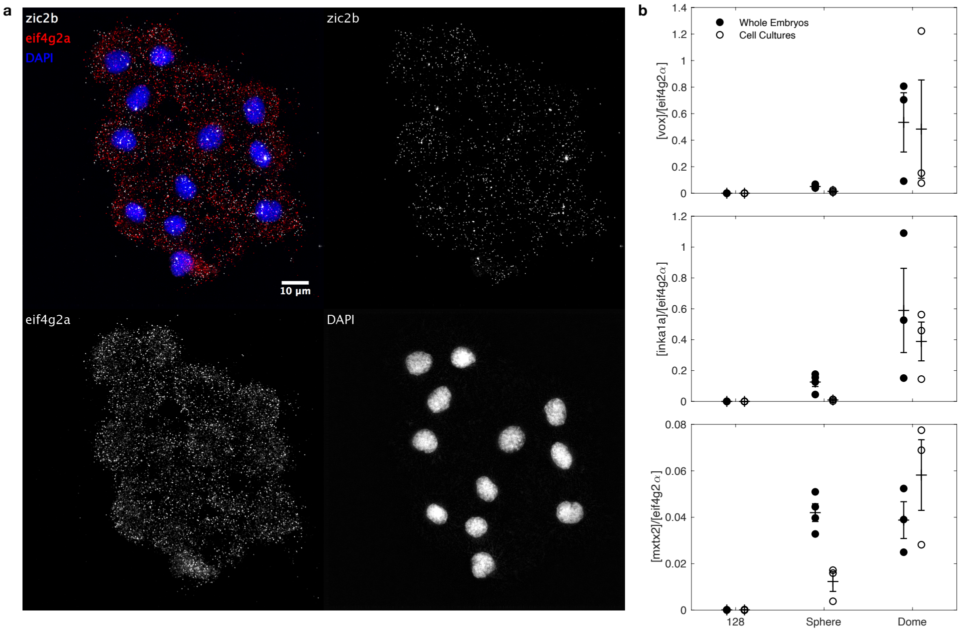

Supplementary Fig. 1

Cells in culture activate transcription in synchrony with cells in the embryo.

a Single molecule RNA FISH for *zic2b* (activated during genome activation), and *eif4g2a* (maternally loaded). Primary cell cultures were obtained at the 512-cell stage and underwent up to 4 cell divisions up to the point of fixation (sphere-equivalent). Microscopy images were recorded using a DeltaVision epifluorescence microscope (60X oil objective, NA 1.42). Experiment was repeated twice with same outcome. **b** Expression of *vox*, *inka1a*, and *mxtx2* (activated during genome activation) was analyzed by qPCR in cultured cells (obtained at the 128-cell stage) and embryos, and normalized to the expression of *eif4g2a* (maternally loaded). Mean±SD, $n = 4,3,4,3,3,3$ experimental repeats in the conditions 128 Whole Embryos, 128 Cell Cultures, Sphere Whole Embryos, Sphere Cell Cultures, Dome Whole Embryos, respectively.

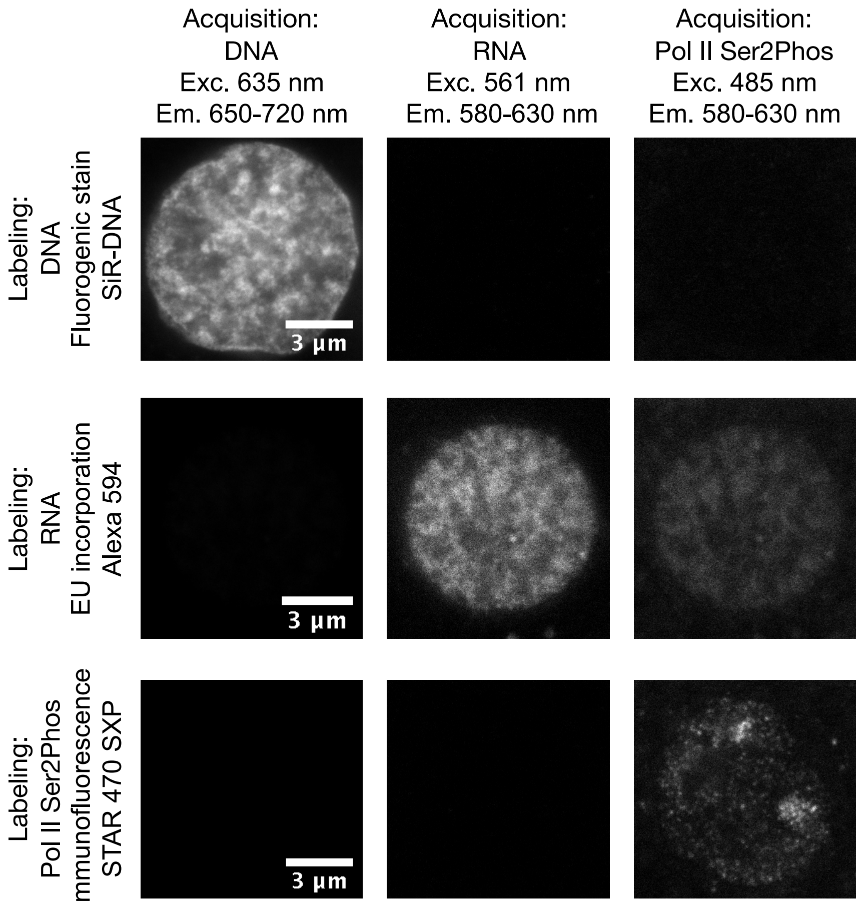

Supplementary Fig. 2

Three-color STED super-resolution microscopy of DNA, RNA, and transcriptional activity.

Three STED super-resolution micrographs from nuclear mid-sections, stained for either DNA, RNA, or transcriptional activity (detected by RNA polymerase II C terminal domain Serine 2 phosphorylation, Pol II Ser2Phos). Each label can be detected in one color channel with negligible crosstalk to the other channels. Representative image, $N = 3$ images were acquired per label.

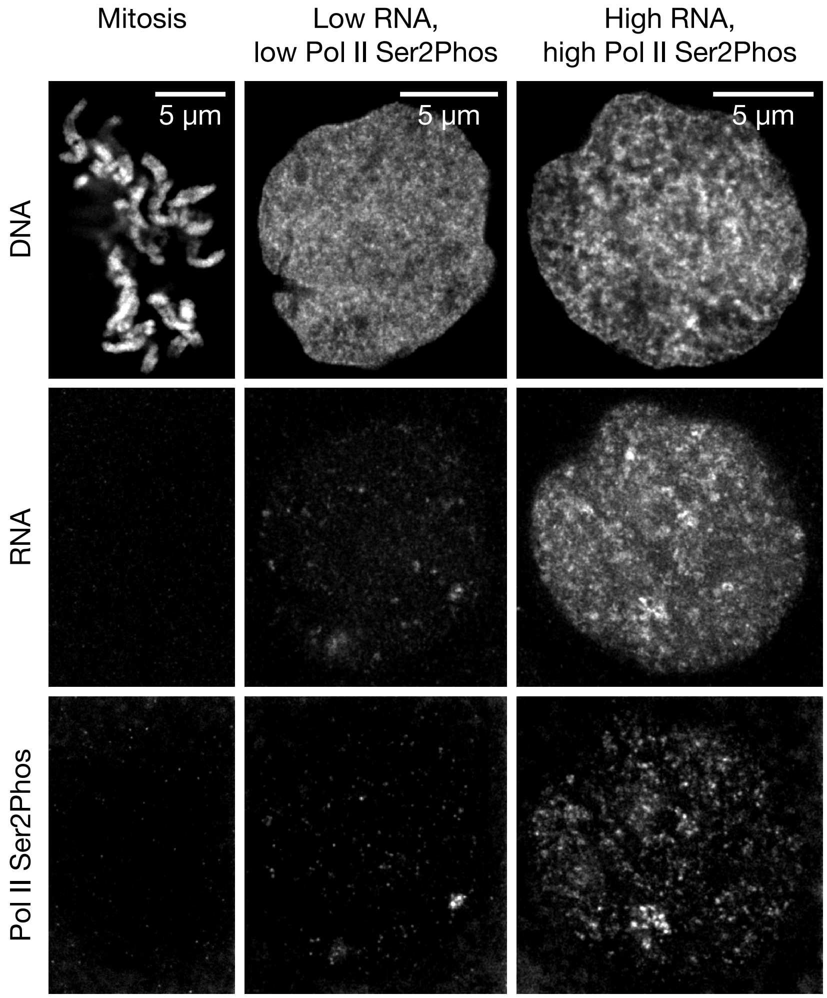

Supplementary Fig. 3

STED super-resolution micrographs of mitotic chromosomes, as well as nuclei before and after transcription onset.

Representative three-color STED super-resolution micrographs showing DNA, RNA, and transcriptional activity (Pol II Ser2Phos) during mitosis (**a-c**), shortly after mitosis (**d-f**), and in interphase after full transcription onset (**g-i**). All nuclei recorded from the same sample; acquisition settings and intensity maps are kept the same across all imaged nuclei. The same results were obtained in 4 independent experiments.

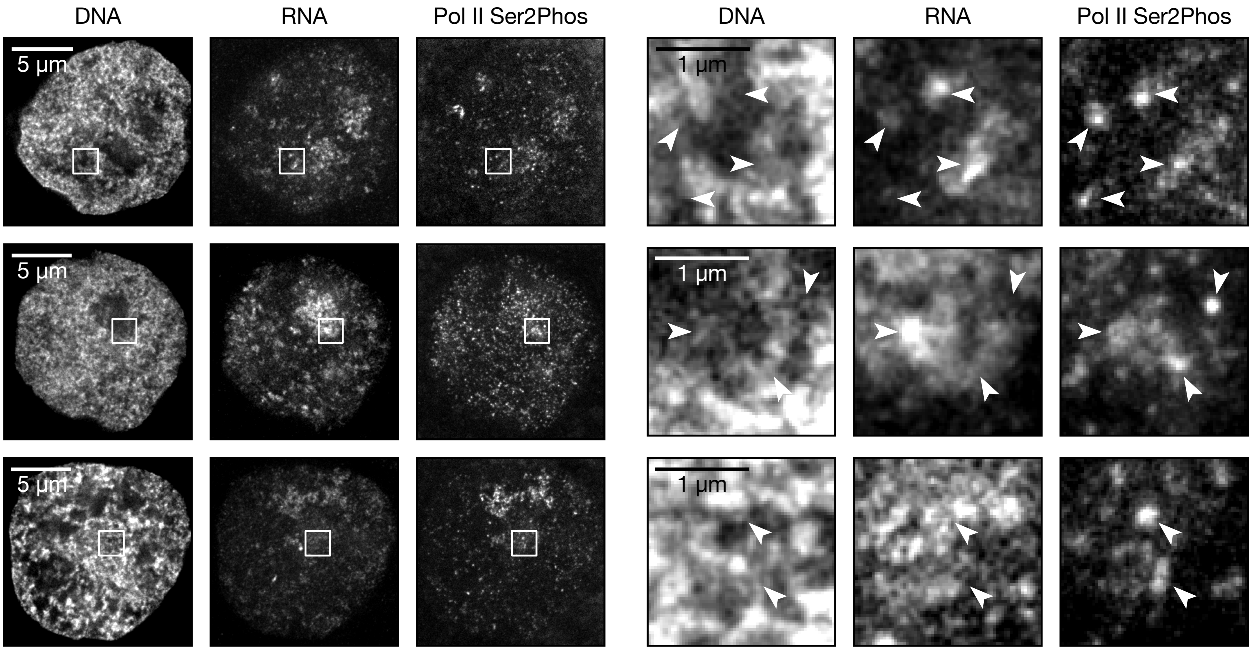

Supplementary Fig. 4

Detailed views of the relative organization of DNA, RNA, and transcriptional activity. Micrographs of nuclear mid-sections from three different nuclei of untreated cell cultures. These images are provided as additional examples of the relative localization of DNA, RNA, and transcriptional activity (Pol II Ser2Phos), as shown in the main manuscript Fig. 1f. Arrows are added for visual support, indicating the same location across the color channels. The same results were obtained in 4 independent experiments.

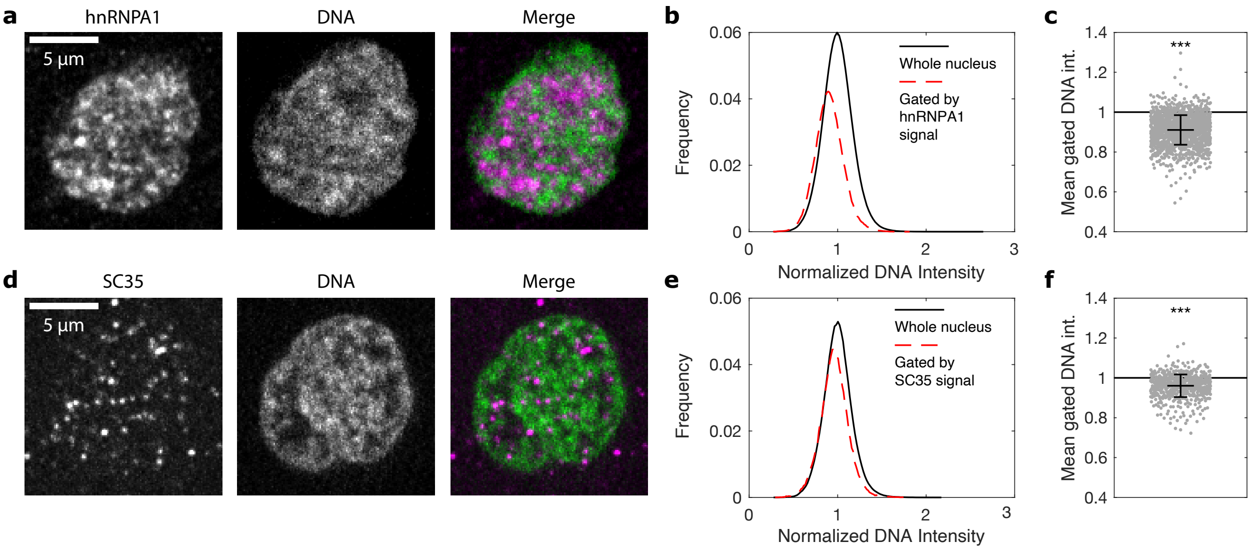

Supplementary Fig. 5

RNA-binding proteins segregate from chromatin.

a Representative nuclear mid-section showing hnRNPA1 and DNA distribution. hnRNPA1 labeled by immunofluorescence, DNA labeled with DAPI, images acquired by spinning disk confocal microscopy. The same results were obtained in 3 independent experiments. **b** Histogram showing distribution of DNA intensities throughout the entire nucleus vs. inside of hnRNPA1 foci. Whole nucleus distribution obtained by segmentation of DAPI signal, gating by hnRNPA1 foci by further segmentation based on the hnRNPA1 channel. Whole nucleus signal was normalized per-nucleus to have a mean of 1, the hnRNPA1-gated pixels have a mean of 0.91. **c** Mean of the normalized DNA intensity after gating by hnRNPA1 signal is lower than the ungated mean DNA intensity (indicated by a line with value 1.0). Mean±SD, *** indicates $P < 0.001$ (P value $< 10^{-5}$ for differences of mean from a two-sided permutation test, $n = 1643$ nuclei from 3 independent experiments). **d** Representative nuclear mid-sections showing SC35 and DNA distribution. SC35 labelled by immunofluorescence. The same results were obtained in 3 independent experiments. **e** Histogram showing distribution of DNA intensities throughout the entire nucleus vs. inside of SC35 foci. The SC35-gated pixels have a mean of 0.96. **f** Mean of the normalized DNA intensity after gating by SC35 signal is lower than the ungated mean DNA. Mean±SD *** indicates $P < 0.001$ (P value $< 10^{-5}$ for differences of mean from a two-sided permutation test, $n = 636$ nuclei from 3 independent experiments).

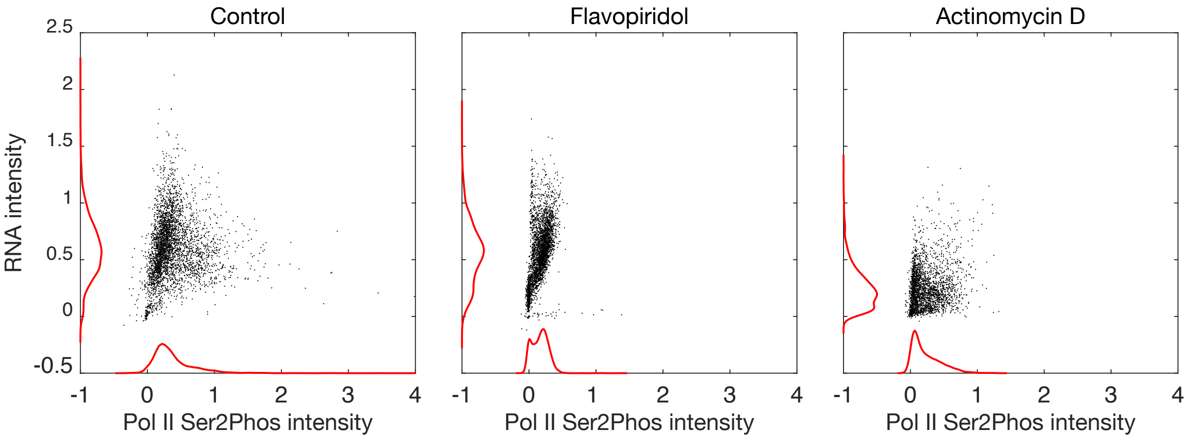

Supplementary Fig. 6

Inhibitor effect on transcriptional activity and RNA levels.

Dissociated cells were cultured in control media, media with flavopiridol, or media with actinomycin D. Transcriptional activity (Pol II Ser2Phos) and RNA accumulation in the cell nucleus were quantified from fixed cells (data points pooled from 3 samples per condition, scaled by 95 percentiles in the control condition of a given sample, subtraction of cytoplasmic background was applied before scaling which can lead to negative values). The different effects of flavopiridol and actinomycin D treatment can be expected based on their mechanisms of action. Flavopiridol prevents the transition of RNA polymerase II from initiation to elongation, but not elongation itself. Thus, elongating RNA polymerase II is lost as the transcription of genes is completed and no new elongation is established. RNA production, however, continues for a considerable length of time, while the elongation of currently transcribed genes is completed. Actinomycin D arrests elongating RNA polymerase II, which then remains bound to DNA and retains the Ser2Phos mark. Thus, inhibition by actinomycin D immediately stops further production of RNA transcripts but only a modest reduction in the Pol II Ser2Phos signal is seen. Plots show $n = 3523, 3398, 2582$ individual nuclei with probability densities from 3 different experiments per condition.

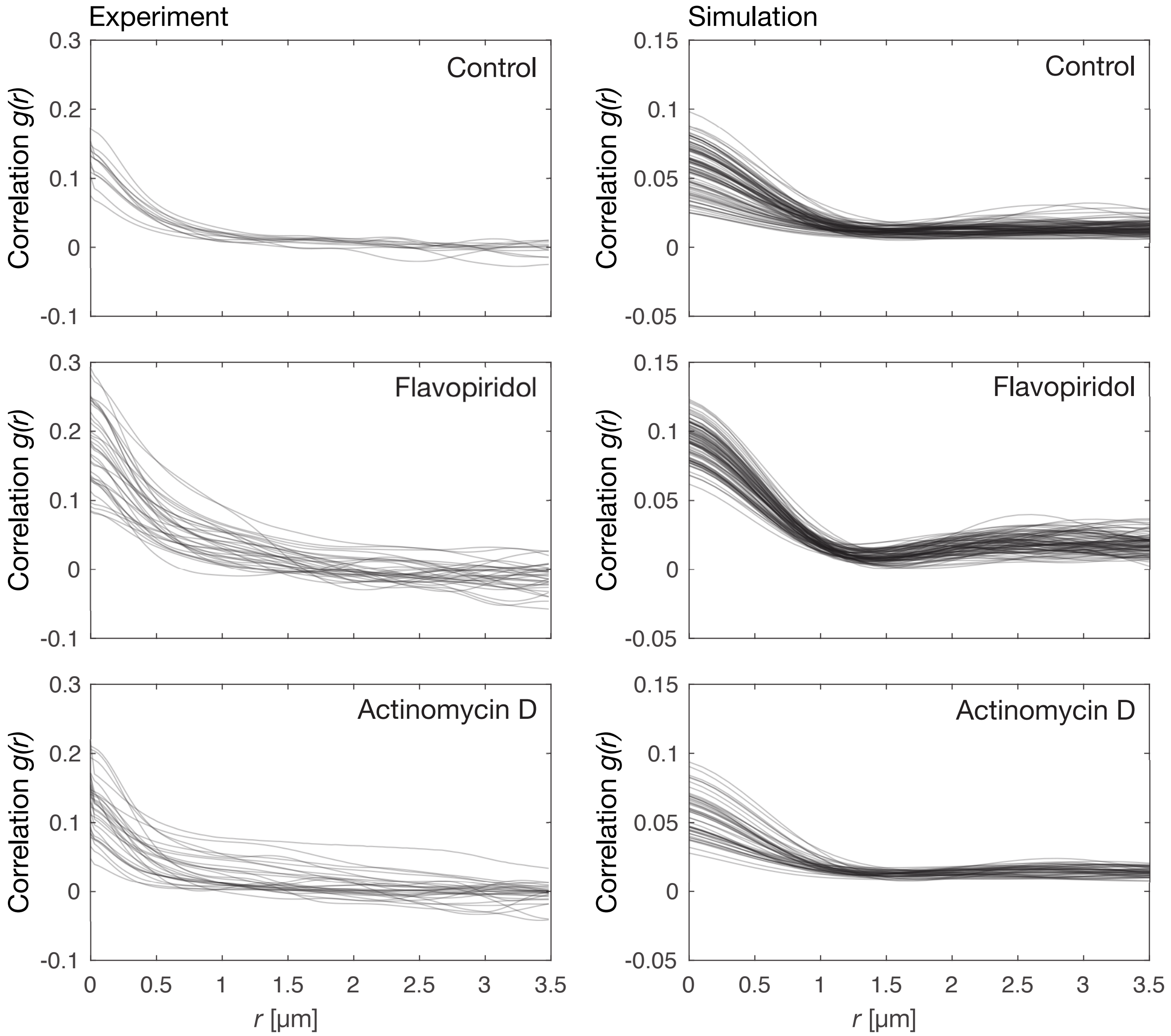

Supplementary Fig. 7

Correlation functions for different inhibitor treatment conditions.

The radial correlation function, $g(r)$, is shown for the different inhibitor treatment conditions shown in the main manuscript. The correlation functions for experiments are based on the DNA intensity profiles inside the nuclei in STED super-resolution nuclear mid-sections. The graphs contain one curve for every nucleus contained in the data set used in the main manuscript (Fig. 2c). The correlation functions for simulations are based on the DNA concentration profiles obtained from lattice simulations. The graphs contain one curve for each simulation that is used in the main manuscript (Fig. 3f).

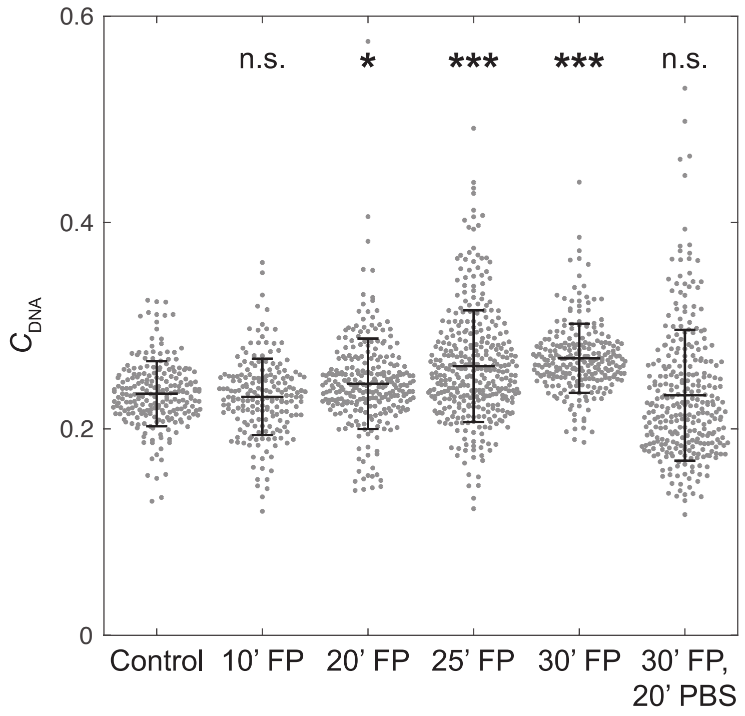

Supplementary Fig. 8

Flavopiridol-induced chromatin coarsening is reversible.

Zebrafish embryonic cells were cultured for a total of 30 minutes, and flavopiridol (FP) was applied for the indicated time leading up to the end of these 30 minutes. The cultures were fixed after treatment and the image contrast in the DNA channel in mid-sections of individual nuclei (C_{DNA}) was quantified from spinning disk confocal microscopy image data. In the condition 30’ FP, 20’ PBS, FP treatment was followed by a 20-minute wash-out period where media was replaced with PBS without flavopiridol. Mean±SD, * indicates $P < 0.05$ for differences in the mean relative to the control condition, *** indicates $P < 0.001$, n.s. indicates $P \geq 0.05$ (P values $1.83, 0.03, < 10^{-5}, < 10^{-5}, 3.74$ from a two-sided permutation test, P values with Bonferroni correction for multiple testing, $n = 214, 192, 269, 342, 239, 300$ nuclei from a single experiment).

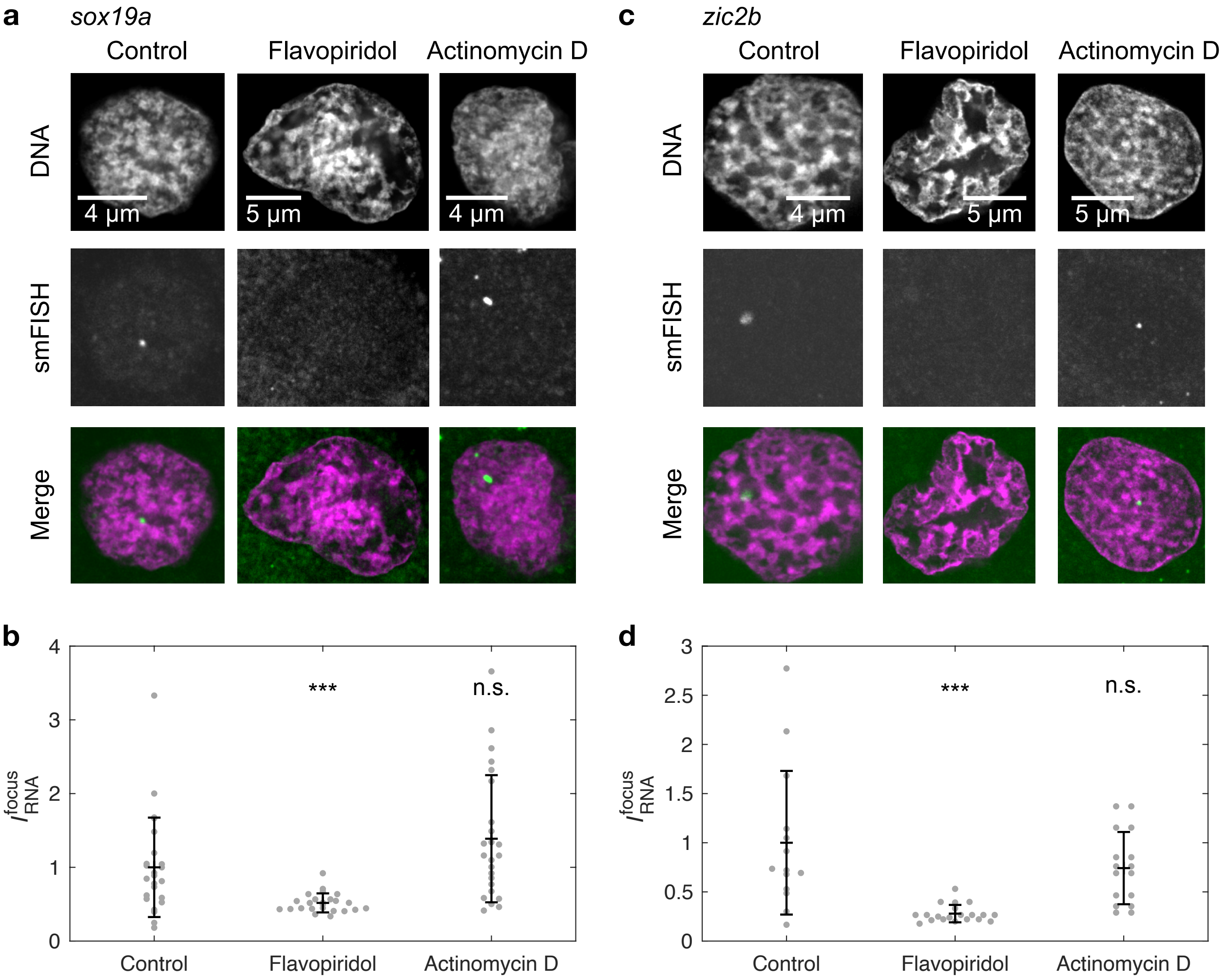

Supplementary Fig. 9

Transcripts are released from chromatin upon flavopiridol treatment but retained upon actinomycin D treatment.

a Representative nuclear mid-sections of fixed zebrafish embryo primary cell cultures after 30 minutes of the indicated treatment. STED super-resolution microscopy of DNA was combined with regular confocal microscopy of the mRNA transcript *sox19a* (labeled with RNA single molecule FISH, smFISH). **b** Quantification of the intensity of the brightest spot that could be detected in a given nuclear mid-section ($I_{\mathrm{RNA}}^{\mathrm{focus}}$). Mean±SD, *** indicates $P < 0.001$ (P values $0.0002, 0.18$ from a two-sided permutation test for differences from Control, P values with Bonferroni correction for multiple testing, $n = 24, 24, 24$ nuclei from 2 different samples per condition). **c** Same as panels A and B, but using an smFISH probe against *zic2b* mRNA. **d** Quantification of the intensity of the brightest spot that could be detected in a given nuclear mid-section. Mean±SD, *** indicates $P < 0.001$, n.s. indicates $P \geq 0.05$ (P values $< 10^{-5}, 0.44$ from a two-sided permutation test, P values with

Bonferroni correction for multiple testing, $n = 24, 24, 24$ nuclei from 2 different samples per condition).

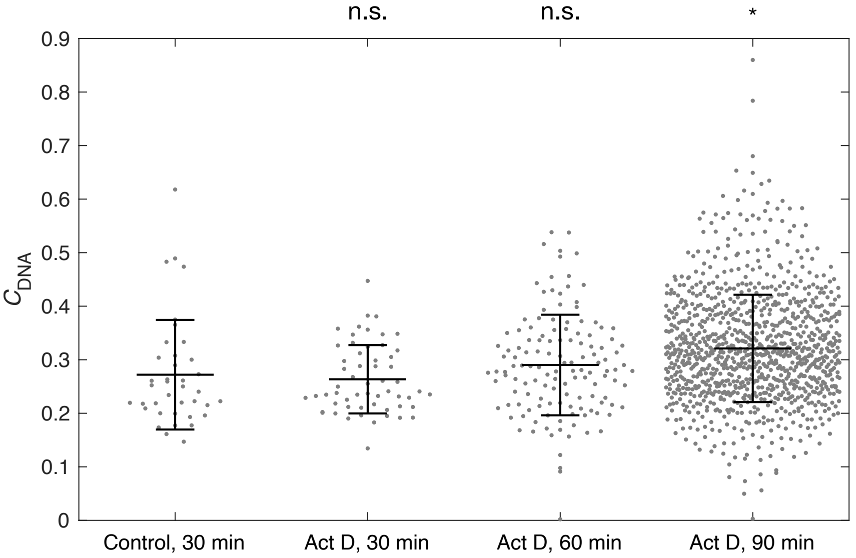

Supplementary Fig. 10

Actinomycin D induces chromatin coarsening only after extended treatment. Dissociated cells were cultured and treated with actinomycin D (Act D) for the indicated duration, then fixed, stained, and imaged by spinning disk confocal microscopy. Mean±SD, ** indicates $P < 0.01$, n.s. indicates $P \geq 0.05$ (P values $1.87, 0.92, 0.01$ from a two-sided permutation test, P values with Bonferroni correction for multiple testing, $n = 38, 57, 133, 1020$ nuclei obtained from a single experiment). The observation of an increased C_{DNA} at 90 minutes of treatment is in line with previous observations of chromatin coarsening after 2 hours of actinomycin D treatment[1].

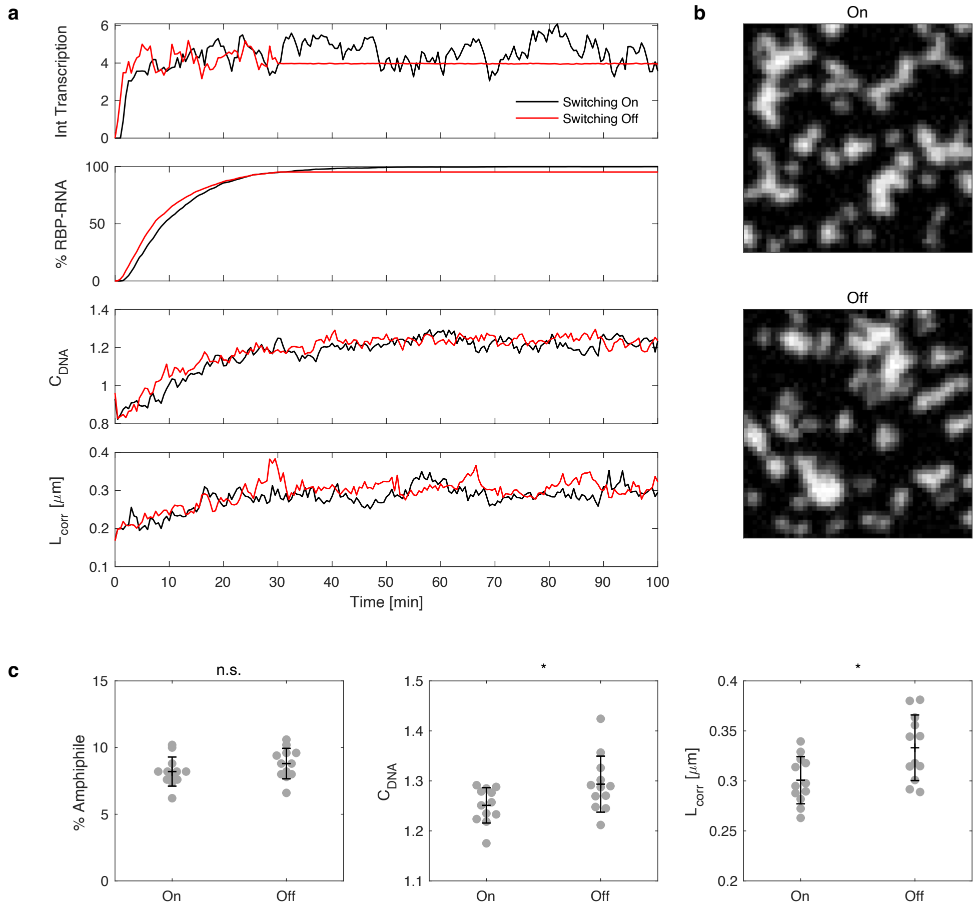

Supplementary Fig. 11

Chromatin microdomains are slightly larger when amphiphiles are kept at static positions on simulated chromatin chains.

In the lattice model simulations, monomers that represent transcription elongation and exert an amphiphile effect are stochastically placed on chromatin chains and subsequently deactivated. Here we assess in how far this stochastic placement-removal cycle affects chromatin organization. **a** Simulations were evaluated where the switching of transcription sites was turned off (Off) after an initial 30 minutes of simulation time. RNA was retained at these transcription sites to produce static amphiphile positions. To retain high levels of RNA that is bound to RBPs despite arresting RNA release from chromatin to RBPs, the RNA degradation rate was set to zero ($k^{-}_{\mathrm{RNA}} = 0$). These simulations were compared with simulations where switching of transcription sites was permitted (On), which were implemented using the parameters of the example case simulations (Supplementary Table 1). In the On case, the RNA production rate was increased to $k^{+}_{\mathrm{RNA}} = 0.1\ \mathrm{s}^{-1}$ to achieve a comparable level of RNA-RBP complex formation as in the Off case (% RBP-RNA).

Lattice size was 50×50 pixels, overall parameters were the same as for the example microemulsion outcomes (Supplementary Table 1). To allow quantification of image contrast (C_{DNA}) and correlation length (L_{corr}), a Gaussian blur filter with standard deviation (σ) of 1 pixel was applied. Note that the transcription level reached a stochastic steady state before reactions were turned off at 30 min. C_{DNA} and L_{corr} reach an apparent stochastic steady state level after 100 minutes simulation time. **b** Simulated DNA concentration profiles obtained at the end of 100 min simulation time. **c** Statistical comparison of percentage of total chromatin labelled as amphiphile particles (% amphiphile), C_{DNA}, and L_{corr}. Mean$\pm$SD, * indicates $P < 0.05$, n.s. indicates $P \geq 0.05$ (P values from two-sided permutation test, $n = 12$ simulations for each scenario, $P = 0.21, 0.04, 0.01$).

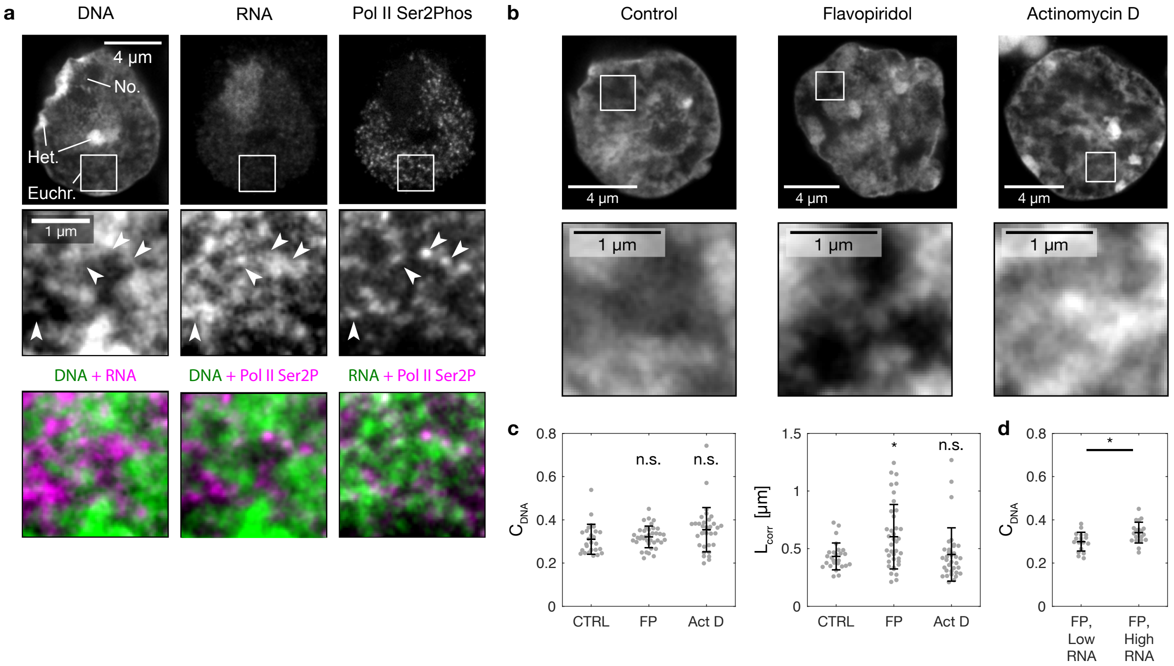

Supplementary Fig. 12

Validation of fundamental microemulsion predictions in mouse embryonic stem cells.

a Representative nuclear mid-section of a fixed mouse embryonic stem cell (mESC). Nucleolus (No.), heterochromatin domains (Het.), and a euchromatic region (Euch.) are indicated. A zoom into part of a euchromatin region is indicated by a white box, arrows indicate sites with high Pol II Ser2Phos signal. Same fluorescence labeling and recording approaches were used as for primary zebrafish embryo cell cultures. The same results were obtained in 4 independent experiments. **b** Representative nuclear mid-sections showing DNA distributions after 30 minutes of treatment as indicated, with zooms into euchromatic regions. The same results were obtained in 3 independent experiments. **c** Quantification of image contrast (C_{DNA}) and correlation length (L_{corr}) in the DNA channel inside euchromatin regions after 30 minutes of inhibitor treatment as indicated (flavopiridol – FP, actinomycin D – Act D). Mean±SD, * indicates $P < 0.05$, n.s. indicates $P \geq 0.05$ (C_{DNA} P values $1.40, 0.18$ from a two-sided permutation test, L_{corr} P values $0.01, 2.23$ from a two-sided permutation test, P values with Bonferroni correction for multiple testing, $n = 26, 38, 34$ nuclei from 6, 6, 5 different samples). **d** Quantification of C_{DNA} in the flavopiridol-treated samples, for low and high RNA levels. Mean±SD, * indicates $P < 0.05$ (P value 0.02 from a two-sided permutation test, $n = 18, 20$ from 6 different samples). All data acquired by STED super-resolution microscopy

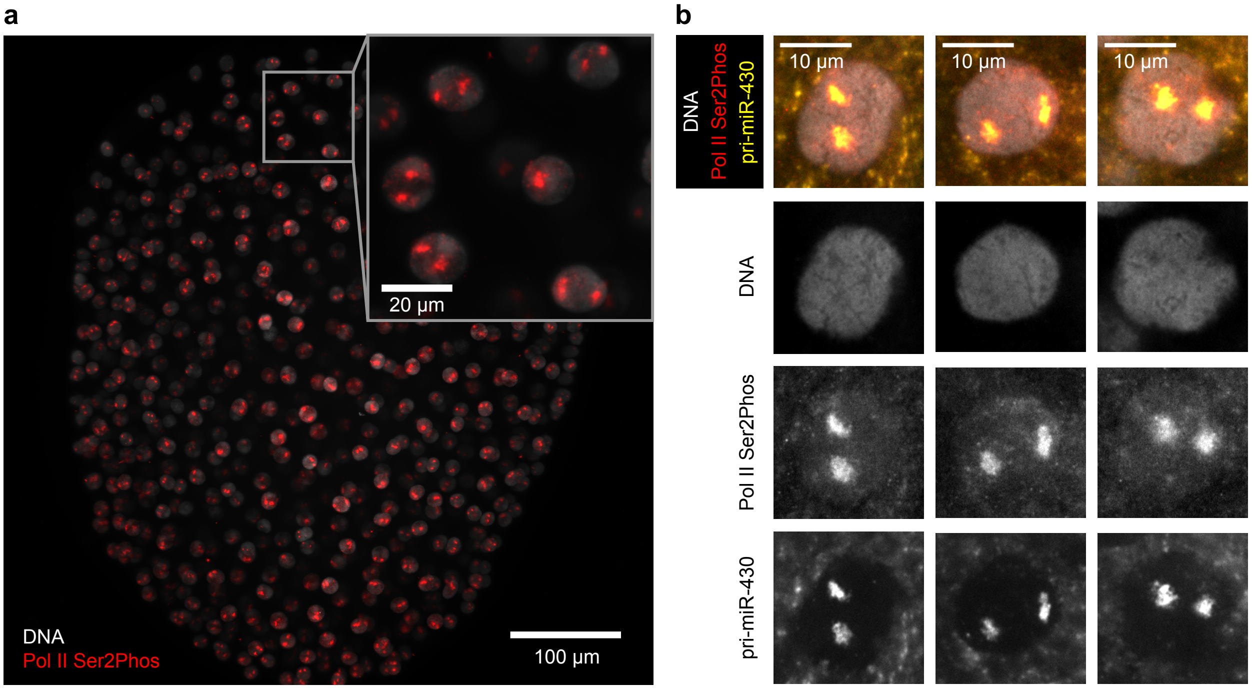

Supplementary Fig. 13

Two prominent transcription foci associated with the miR-430 gene cluster in nuclei of late blastula zebrafish embryos.

a Light sheet micrograph showing the occurrence of two prominent transcription sites (Pol II Ser2Phos) in the nuclei (DNA labelled with DAPI) of a fixed late blastula stage zebrafish embryo (animal view, maximum intensity projection). The same results were obtained in 2 independent experiments. **b** Three example micrographs showing the colocalization of the two prominent transcription sites with miR-430 primary transcripts (pri-miRNA, revealed by fluorescence *in situ* hybridization, FISH) (probes provided by van Boxtel and colleagues[2]). Images acquired by spinning disk confocal microscopy, shown are single, mid-nuclear optical sections of three representative nuclei. Note that the FISH procedure can perturb the fine structure of chromatin, so that the depletion of DNA at the transcription sites is not as obvious as in images shown in other figures. The same results were obtained in 2 independent experiments.

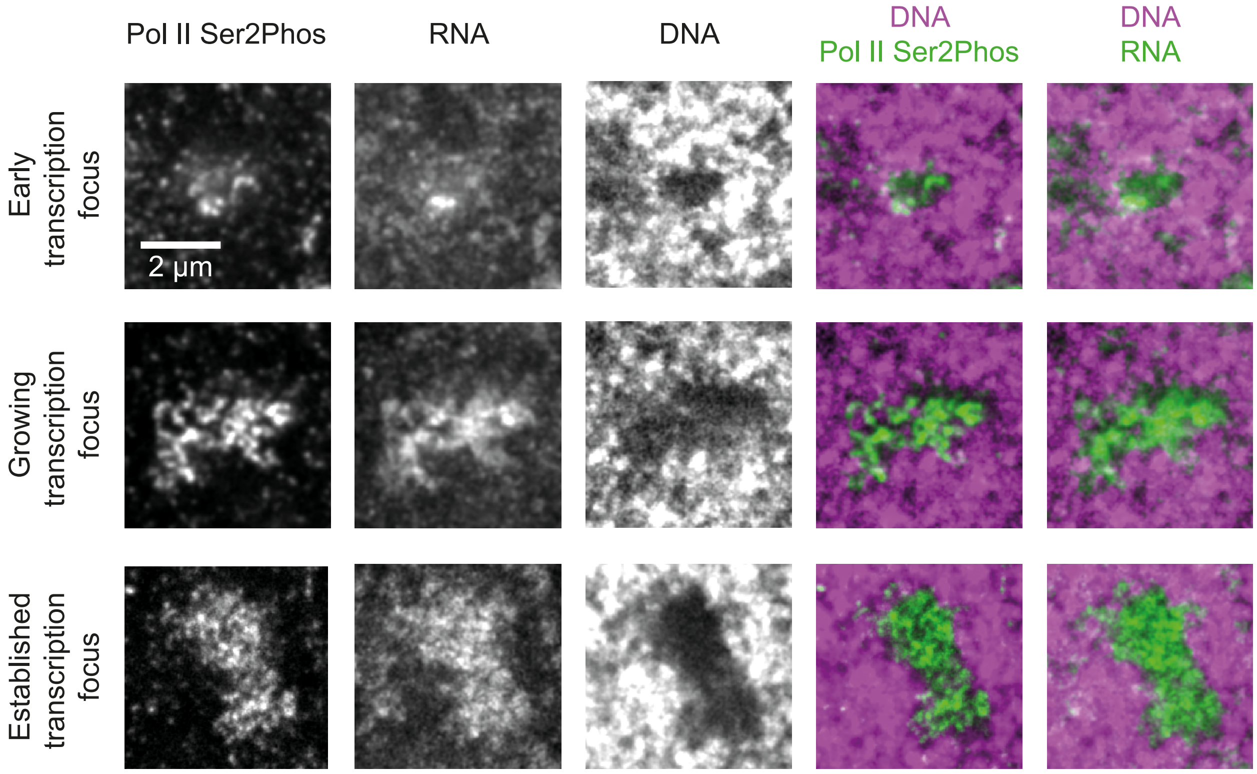

Supplementary Fig. 14

Super-resolution assessment of the establishment of prominent transcription foci. Representative zooms into STED super-resolution mid-sections of nuclei of fixed zebrafish cells, showing prominent transcription foci in different stages of their emergence. As predicted by our simulations of transcription onset at isolated sites, RNA accumulates in the vicinity of transcription sites, and DNA is displaced from RNA-filled regions. The same results were obtained in 2 independent experiments.

Simulation of example microemulsion outcomes		
Repulsion strength[1]	w	$0.5k_BT$
Swapping rate[2]	s	$3{,}000\ 1/s$
Transcription on rate[2,*]	k_{chrom}^{+}	$0.001\ 1/s$
Transcription off rate[2,*]	k_{chrom}^{-}	$0.01\ 1/s$
RNA production rate[2,*]	k_{RNA}^{+}	$0.01\ 1/s$
RNA decay rate[2,*]	k_{RNA}^{-}	$0.0001\ 1/s$
RNA transfer rate[2,*]	$k_{\mathrm{RNA}}^{transfer}$	$0.01\ 1/s$
**Simulation of inhibitor treatment experiments**		
Repulsion strength[1]	w	$0.5k_BT$
Swapping rate[3]	s	$10{,}000\ 1/s$
Chain on rate[4]	$k_{\mathrm{chain}}^{\mathrm{on}}$	$0.00025\ 1/s$
Chain off rate[4]	$k_{\mathrm{chain}}^{\mathrm{off}}$	$0.0033\ 1/s$
Transcription on rate[5]	k_{chrom}^{+}	$0.005\ 1/s$
Transcription off rate[6]	k_{chrom}^{-}	$0.0017\ 1/s$
RNA production rate[7]	k_{RNA}^{+}	$0.1\ 1/s$
RNA decay rate[8]	k_{RNA}^{-}	$0.00083\ 1/s$
RNA transfer rate[9]	$k_{\mathrm{RNA}}^{\mathrm{transfer}}$	$0.0033\ 1/s$
**Simulation of miR-430 transcription onset**		
Repulsion strength[1]	w	$0.5k_BT$
Swapping rate[3]	s	$10{,}000\ 1/s$
Chain on rate[10]	$k_{\mathrm{chain}}^{\mathrm{on}}$	$1/s$
Chain off rate[10]	$k_{\mathrm{chain}}^{\mathrm{off}}$	0
Transcription on rate[10]	k_{chrom}^{+}	$0.0083\ 1/s$
Transcription off rate[10]	k_{chrom}^{-}	0
RNA production rate[7]	k_{RNA}^{+}	$0.1\ 1/s$
RNA decay rate[8]	k_{RNA}^{-}	$0.00083\ 1/s$
RNA transfer rate[9]	$k_{\mathrm{RNA}}^{\mathrm{transfer}}$	$0.0033\ 1/s$

Supplementary Table 1: Model parameters. [1]Minimally required value for phase separation[3], larger values slowed down the dynamics in our simulation. [2]Generic values chosen for demonstration purposes. *These parameters only take effect in the microphase separation scenario, not the dispersion and the phase separation scenario. [3]Adjusted to achieve relaxation of free chromatin chains within 10 min. [4]Chosen so that every chain activates approximately once per cell cycle, and then turns off again within 5 min. [5]Rate of Pol II escape from paused state[4]. [6]Average time of 10 min for transcript completion estimated based on a typical length of genes transcribed at late blastula stage of 10 kb[5] and a typical Pol II transcription rate of 1 kb/min[6]. [7]Arbitrarily chosen, as it can be scaled into absolute RNA concentration. [8]Within the range of typical nuclear retention times of completed transcripts[7,8]. [9]Chosen as half of k_{chrom}^{-}, so as to reflect the release of first complete RNA transcripts while other transcripts are still being synthesized. [10]Adjusted to

reflect the faster and sustained transcriptional induction of the miR-430 locus relative to other genes that are modelled in the inhibitor experiment simulations.

**Gene**	**Primers**
vox[1]	5'-TTATTCGTCGGGTTATGAGAG
	5'-AACCAAGTTCTGATCTGTGT
inka1a[1] (previously *fam212aa*)	5'-GCAAATGAGTATCTAAAACTGCT
	5'-CATCATATAGCGCATCTGGT
mxtx2[1]	5'-ACTGACTGCATTGCTCAA
	5'-ACCATACCTGAATACGTGATT
eif4g2a[1]	5'-GAGATGTATGCCACTGATGAT
	5'-GCGCAGTAACATTCCTTTAG

Supplementary Table 2: Primers. [1]Used previously by Joseph and colleagues[9].

Supplementary References

1. Nickerson, J. A., Krochmalnic, G., Wan, K. M. & Penman, S. Chromatin architecture and nuclear RNA. *Proc. Natl. Acad. Sci. USA* **86**, 177–181 (1989).
2. van Boxtel, A. L. *et al.* A Temporal Window for Signal Activation Dictates the Dimensions of a Nodal Signaling Domain. *Dev. Cell* **35**, 175–185 (2015).
3. Larson, R. G., Scriven, L. E. & Davis, H. T. Monte Carlo simulation of model amphiphile-oil–water systems. *J. Chem. Phys.* **83**, 2411–2420 (1985).
4. Stasevich, T. J. *et al.* Regulation of RNA polymerase II activation by histone acetylation in single living cells. *Nature* **516**, 272–275 (2014).
5. Heyn, P. *et al.* The earliest transcribed zygotic genes are short, newly evolved, and different across species. *Cell Rep.* **6**, 285–292 (2014).
6. Jonkers, I., Kwak, H. & Lis, J. T. Genome-wide dynamics of Pol II elongation and its interplay with promoter proximal pausing, chromatin, and exons. *Elife* **3**, e02407 (2014).
7. Bahar Halpern, K. *et al.* Nuclear Retention of mRNA in Mammalian Tissues. *Cell Rep.* **13**, 2653–2662 (2015).
8. Battich, N., Stoeger, T. & Pelkmans, L. Control of Transcript Variability in Single Mammalian Cells. *Cell* **163**, 1596–1610 (2015).
9. Joseph, S. R. *et al.* Competition between histone and transcription factor binding regulates the onset of transcription in zebrafish embryos. *Elife* **6**, e23326 (2017).
