# Supplementary material for: Transcription organizes euchromatin via microphase separation
Source: Nat Commun. 2021 Mar 1;12:1360. doi: 10.1038/s41467-021-21589-3 (PMC7921102; doi:10.1038/s41467-021-21589-3)
Supplement: Supplementary file 2 — Reporting Summary [file 41467_2021_21589_MOESM2_ESM.pdf]

nature research

Corresponding author(s): Nadine Vastenhouw
Last updated by author(s): Jan 22, 2021

Reporting Summary

Nature Research wishes to improve the reproducibility of the work that we publish. This form provides structure for consistency and transparency in reporting. For further information on Nature Research policies, see our Editorial Policies and the Editorial Policy Checklist.

Statistics

For all statistical analyses, confirm that the following items are present in the figure legend, table legend, main text, or Methods section.

n/a	Confirmed	
☐	☒	The exact sample size (n) for each experimental group/condition, given as a discrete number and unit of measurement
☐	☒	A statement on whether measurements were taken from distinct samples or whether the same sample was measured repeatedly
☐	☒	The statistical test(s) used AND whether they are one- or two-sided<br>*Only common tests should be described solely by name; describe more complex techniques in the Methods section.*
☐	☒	A description of all covariates tested
☐	☒	A description of any assumptions or corrections, such as tests of normality and adjustment for multiple comparisons
☐	☒	A full description of the statistical parameters including central tendency (e.g. means) or other basic estimates (e.g. regression coefficient) AND variation (e.g. standard deviation) or associated estimates of uncertainty (e.g. confidence intervals)
☐	☒	For null hypothesis testing, the test statistic (e.g. F, t, r) with confidence intervals, effect sizes, degrees of freedom and P value noted<br>*Give P values as exact values whenever suitable.*
☒	☐	For Bayesian analysis, information on the choice of priors and Markov chain Monte Carlo settings
☒	☐	For hierarchical and complex designs, identification of the appropriate level for tests and full reporting of outcomes
☒	☐	Estimates of effect sizes (e.g. Cohen's d, Pearson's r), indicating how they were calculated

Our web collection on statistics for biologists contains articles on many of the points above.

Software and code

Policy information about availability of computer code

Data collection	Simulation results were produced using a custom-developed C++ core library with Python- and bash-based utility functions, all of which are hosted and documented as a GitHub open source repository (github.com/lhilbert/active-microemulsion).
Data analysis	The microscopy data were analyzed using MatLab (R2018b, 9.5.0.944444), relying on the import function provided in the bioformats importer toolbox. The working steps implemented in the form of MatLab scripts are standard image analysis procedures, and are described in detail in the Supplementary Methods. Representative micrographs were digitally generated using MatLab and FIJI.

For manuscripts utilizing custom algorithms or software that are central to the research but not yet described in published literature, software must be made available to editors and reviewers. We strongly encourage code deposition in a community repository (e.g. GitHub). See the Nature Research guidelines for submitting code & software for further information.

Data

Policy information about availability of data

All manuscripts must include a data availability statement. This statement should provide the following information, where applicable:
- Accession codes, unique identifiers, or web links for publicly available datasets
- A list of figures that have associated raw data
- A description of any restrictions on data availability

All relevant data are available from the authors upon request.

Field-specific reporting

Please select the one below that is the best fit for your research. If you are not sure, read the appropriate sections before making your selection.

- [x] Life sciences
- [ ] Behavioural & social sciences
- [ ] Ecological, evolutionary & environmental sciences

For a reference copy of the document with all sections, see nature.com/documents/nr-reporting-summary-flat.pdf

Life sciences study design

All studies must disclose on these points even when the disclosure is negative.

Sample size	No statistical methods were used to predetermine sample size.<br><br>In spinning disk confocal microscopy experiments, we routinely obtained tens to hundreds of measurement points from a single sample. A combination of data from any two out of these three samples displayed the same overall distribution, so that we assumed that three samples were sufficient to capture the distribution of data. In STED super-resolution microscopy experiments, we collected data until the distributions of transcription and RNA intensities approximated the distributions obtained from spinning disk microscopy. Under these conditions, we assumed that the STED data sufficiently covered the distributions determined by spinning disk microscopy.<br><br>For Supplementary Figs. 8 and 10, all data points were drawn from a single sample per condition. Here, already several tens of nuclei per condition were obtained, and statistical power was evidenced by conditions with $p<0.001$.<br><br>For Supplementary Fig. 9 two independent samples per condition, not three were acquired. Instead, the experiment was carried out with probes for two different transcripts, which both gave statistically significant and completely identical results. This approach seemed better than a higher number of repeats with a single probe.<br><br>For Supplementary Fig. 12, the number of cell nuclei recorded from mouse embryonic stem cells was adjusted so that it was comparable to the number of nuclei recorded from zebrafish cells.
Data exclusions	The fluorescence labeling of fixed samples exhibited the common variability in staining intensity, and poorly labeled samples could not be analyzed. Some biological samples were accidentally lost or damaged during processing, and in consequence could not be included in data acquisition. All other experimental results were included in the analysis.
Replication	All attempts at replication were successful.
Randomization	No animal or patient groups were used in our study, hence no randomization was required.
Blinding	Investigators were not blinded to group allocation. Where experimental designs did not include different groups, conditions, or treatments, no blinding is required. Where samples from different conditions were compared, the data acquisition was organized in a way to capture images from the overall sample in an unbiased manner, and analysis was carried out using automated analysis scripts, so that investigator bias is prevented during data acquisition and processing.

Reporting for specific materials, systems and methods

We require information from authors about some types of materials, experimental systems and methods used in many studies. Here, indicate whether each material, system or method listed is relevant to your study. If you are not sure if a list item applies to your research, read the appropriate section before selecting a response.

Materials & experimental systems

n/a	Involved in the study	
	x	Antibodies
	x	Eukaryotic cell lines
x		Palaeontology and archaeology
	x	Animals and other organisms
x		Human research participants
x		Clinical data
x		Dual use research of concern

Methods

n/a	Involved in the study	
x		ChIP-seq
x		Flow cytometry
x		MRI-based neuroimaging

Antibodies

Antibodies used	Primary antibodies:<br>[1] Mouse IgM anti-Pol II CTD Ser2Phos (H5), monoclonal, ab24758 abcam<br>[2] Rabbit IgG anti-Pol II CTD Ser2Phos, monoclonal, ab193468 abcam

[3] Rat IgG anti-H3 Ser28Phos, monoclonal, ab10543, abcam
[4] Mouse IgG SC35, monoclonal, 556363 BD Biosciences
[5] Mouse IgG anti-hnRNPA1, gift from the Black laboratory, not commercially available

Secondary antibodies:
[6] Sheep IgG Anti-Digoxigenin Fab fragments, conjugated with horseradish peroxidase, 1207733 Roche; Dilution: 1:500 for fluorescence in situ hybridization
[7] Goat anti-mouse IgM, conjugated with Alexa 488, A21042 Thermo Fisher
[8] Goat anti-rabbit IgG, conjugated with STAR 470 SXP, 2-0012-008-9, Abberior
[9] Donkey anti-rabbit IgG, conjugated with Alexa 488, A21206 Thermo Fisher
[10] Donkey anti-rat IgG, conjugated with Alexa 488, A21208 Thermo Fisher
[11] Goat anti-rat IgG, conjugated with Alexa 647, A21247 Thermo Fisher

Validation

Primary antibodies:
[1] This antibody clone (H5) was characterized as the main monoclonal antibody to recognize the serine 2 phosphorylation of the RNA polymerase II (Pol II) subunit 1 C-terminal domain heptad repeat (Patturajan, M. et al. 1998, Growth-related changes in phosphorylation of yeast RNA polymerase II, Journal of Biological Chemistry, 273(8), 4689–4694).The Pol II subunit 1 C-terminal domain heptad repeat is highly conserved in eukaryotes, and our own validation in zebrafish cells by immunofluorescence showed the expected distribution, the expected signal loss upon flavopiridol treatment, and overlap with ab193468 antibody in dual-color immunofluorescence.
[2] Supplier validation - human cell lysate: immunoprecipitation-Western Blot, phosphatase treatment, chromatin-immunoPrecipitation-qPCR; human cell culture: immunofluorescence; mouse cell lysate immunoprecipitatioin-Western Blot, phosphatase treatment; mouse tissue section immunohistochemistry; rat cell culture - immunofluorescence; phospho-peptide dot blot assessment of phosphospecificity; the Pol II subunit 1 C-terminal domain heptad repeat is highly conserved in eukaryotes, and own validation in zebrafish cells by immunofluorescence showed the expected distribution, the expected signal loss upon flavopiridol treatment, and overlap with H5 antibody in dual-color immunofluorescence.
[3] Supplier validation - human cell lysate: Western Blot; human cell culture: immunofluorescence; mouse cell culture: immunofluorescence; own validation in zebrafish cells: only nuclei in prophase and prometaphase exhibited immunofluorescence signal.
[4] This antibody was characterized as the main monoclonal antibody to recognize the SC35 protein (Fu, X.-D. and Maniatis, T. 1990, Factor required for mammalian spliceosome assembly is localized to discrete regions in the nucleus, Nature, 343, 437–441) Supplier validation - human cell lysate: Western blot; SC35 is highly conserved in metazoans, own validation in zebrafish cells by immunofluorescence showed the expected distribution.
[5] Validation in previous publication by Western blot and morpholino-mediated target protein depletion in zebrafish larvae (Despic, V. et al. 2017, Dynamic RNA-protein interactions underlie the zebrafish maternal-to-zygotic transition, Genome Research, 27(7), 1184–1194)

Secondary antibodies:
[6-11] Selectivity confirmed by staining without primary antibodies or target hybridization probe, crosstalk assessed by staining with single secondary antibody.

Eukaryotic cell lines

Policy information about cell lines

Cell line source(s)

R1/E murine ES-cells are early passages of subclones from R1 originals (Toronto). These ES cells were established in August 1991 from a male blastocyst hybrid of two 129 substrains (129X1/SvJ and 129S1/SV-+p+Tyr-c Kitl Sl-J/+) and subcloned from Kristina Nagy (former Vintersten) at the European Molecular Biology Laboratory (Heidelberg) in 1998.

Authentication

The cell line is not commercially available and was not authenticated.

Mycoplasma contamination

Cell lines were not specifically tested mycoplasma contamination.

Commonly misidentified lines
(See ICLAC register)

None

Animals and other organisms

Policy information about studies involving animals; ARRIVE guidelines recommended for reporting animal research

Laboratory animals

The study used embryos of Wild Type ABTL zebrafish. No embryos older than 5 days were used, and in accordance with regulation these experiments are not formally considered animal experiments. Parental wild type fish were maintained according to local husbandry regulation, which equally does not formally constitute animal experimentation. To obtain embryos, a male and a female fish were placed in a water tank with a separating net in the afternoon; the fish were placed together the following morning and embryos could be collected after 15 minutes of spontaneous mating. Adult fish (age 6-18 months) were used in this procedure.

Wild animals

No wild animals were used in the study.

Field-collected samples

No field-collected samples were used in the study.

Ethics oversight

Identify the organization(s) that approved or provided guidance on the study protocol, OR state that no ethical approval or guidance was required and explain why not.

Note that full information on the approval of the study protocol must also be provided in the manuscript.
